# Mitochondria-targeted spin-labelled luminescent iridium anticancer complexes†
†Electronic supplementary information (ESI) available. CCDC 1522104. For ESI and crystallographic data in CIF or other electronic format see DOI: 10.1039/c7sc03216a


**DOI:** 10.1039/c7sc03216a

**Published:** 2017-10-20

**Authors:** V. Venkatesh, Raul Berrocal-Martin, Christopher J. Wedge, Isolda Romero-Canelón, Carlos Sanchez-Cano, Ji-Inn Song, James P. C. Coverdale, Pingyu Zhang, Guy J. Clarkson, Abraha Habtemariam, Steven W. Magennis, Robert J. Deeth, Peter J. Sadler

**Affiliations:** a Department of Chemistry , University of Warwick , Coventry CV4 7AL , UK . Email: P.J.Sadler@warwick.ac.uk; b School of Chemistry , WestCHEM , University of Glasgow , Glasgow G12 8QQ , UK . Email: Steven.Magennis@glasgow.ac.uk; c Department of Chemical Sciences , University of Huddersfield , Huddersfield HD1 3DH , UK . Email: c.wedge@hud.ac.uk; d Department of Inorganic and Physical Chemistry , Indian Institute of Science , Bangalore-560012 , India; e School of Pharmacy , University of Birmingham , Edgbaston B15 2TT , UK

## Abstract

Mitochondria generate energy but malfunction in many cancer cells, hence targeting mitochondrial metabolism is a promising approach for cancer therapy. Here we have designed cyclometallated iridium(iii) complexes, containing one TEMPO (2,2,6,6-tetramethylpiperidine-1-oxyl) spin label [C_43_H_43_N_6_O_2_Ir_1_·PF_6_]˙ (**Ir-TEMPO1**) and two TEMPO spin labels [C_52_H_58_N_8_O_4_Ir_1_·PF_6_]˙ (**Ir-TEMPO2**). Electron paramagnetic resonance (EPR) spectroscopy revealed spin–spin interactions between the TEMPO units in **Ir-TEMPO2**. Both **Ir-TEMPO1** and **Ir-TEMPO2** showed bright luminescence with long lifetimes (*ca.* 35–160 ns); while **Ir-TEMPO1** displayed monoexponential decay kinetics, the biexponential decays measured for **Ir-TEMPO2** indicated the presence of more than one energetically-accessible conformation. This observation was further supported by density functional theory (DFT) calculations. The antiproliferative activity of **Ir-TEMPO2** towards a range of cancer cells was much greater than that of **Ir-TEMPO1**, and also the antioxidant activity of **Ir-TEMPO2** is much higher against A2780 ovarian cancer cells when compared with **Ir-TEMPO1**. Most notably **Ir-TEMPO2** was particularly potent towards PC3 human prostate cancer cells (IC_50_ = 0.53 μM), being *ca.* 8× more active than the clinical drug cisplatin, and *ca.* 15× more selective towards cancer cells *versus* normal cells. Confocal microscopy showed that both **Ir-TEMPO1** and **Ir-TEMPO2** localise in the mitochondria of cancer cells.

## Abstract 2

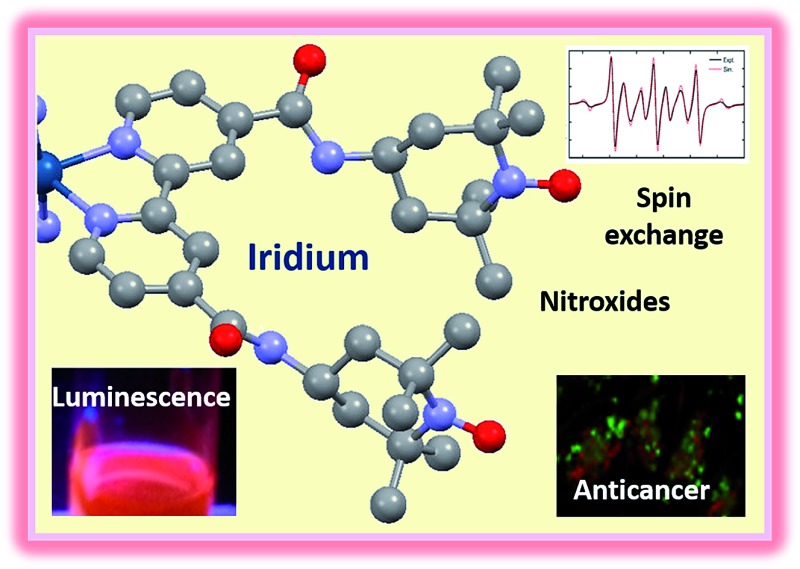
Mitochondria generate energy but malfunction in many cancer cells, hence targeting mitochondrial metabolism is a promising approach for cancer therapy.

## Introduction

The clinical success of platinum complexes has aroused wider interest in the design of metal-based anticancer drugs. The major drawbacks of platinum drugs are their systemic toxicities and development of resistance, now a clinical problem.^1^ New metallodrugs that have different mechanisms of action are needed. Recently we have explored the anticancer activity of organometallic half-sandwich cyclopentadienyl iridium complexes.^2^ They exert anticancer activity by mechanisms which include DNA binding, induction of reactive oxygen species (ROS), and catalytic oxidation.^3^–^5^ In an interesting study, Wilbuer *et al.* reported organometallic iridium complexes that inhibit protein kinase activity,^6^ and recently Mao's group reported the anticancer activity of cyclometallated iridium complexes.^7^,^8^ They took advantage of the interesting photophysical properties of cyclometallated iridium complexes to study their sub-cellular targets. Their bright luminescence with high quantum yields, together with potent anticancer activity, makes this class of compounds potential theranostic agents. The same group utilised cyclometallated iridium complexes as photosensitisers for pH-dependent singlet oxygen production.^9^

Nitroxides are stable free radicals, extensively used as spin labels in electron paramagnetic resonance (EPR) spectroscopy.^10^ Nitroxides have unique antioxidant properties that mimic superoxide dismutase (SOD) and catalase.^11^ The antiproliferative activity of 4-hydroxy/amino-2,2,6,6-tetramethylpiperidine-*N*-oxyl (TEMPO) and their metal complexes is mainly due to the induction of apoptosis through activation of multiple caspases and their activity has been demonstrated in various cancerous and non-cancerous cell lines.^12^–^20^ Nitroxides can inhibit the growth of cancer cells selectively and so are attractive candidates for chemotherapeutic drug design. Substituted nitroxides can selectively target specific sub-cellular compartments, for example mito-TEMPO has a nitroxide attached to triphenylphosphonium chloride that selectively localises in mitochondria and traps ROS.^21^ Mitochondria play crucial roles in many important biological processes, including ATP production, calcium homeostasis, and redox signalling. Mitochondrial dysfunctions are involved in many pathological diseases such as cancer, cardiovascular and neurodegenerative diseases. There is growing interest in targeting mitochondria in cancer cells with therapeutic intervention since they are known to be defective in their function.^22^

**Fig. 1:**
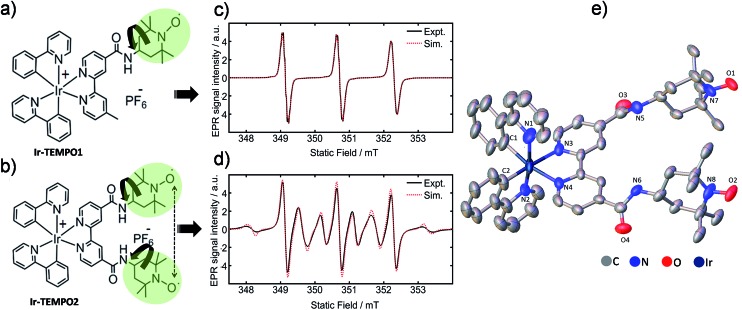
(a and b) Molecular structures of **Ir-TEMPO1** (top) and **Ir-TEMPO2** (bottom); (c and d) X-band EPR spectra of **Ir-TEMPO1** and **Ir-TEMPO2**, as 0.1 mM solutions in deoxygenated DCM. Spectral simulations were performed using EasySpin (see ESI† for details); (e) X-ray crystal structure of **Ir-TEMPO2** showing the close proximity of the two nitroxide radicals (N–N distance 8.63 Å). Hydrogen atoms and counter anion are omitted for clarity, and thermal ellipsoids are drawn at 50% probability.

Here we report the design of novel mono and bis TEMPO (2,2,6,6-tetramethylpiperidine-*N*-oxyl)-substituted cyclometallated iridium complexes (**Ir-TEMPO1** and **Ir-TEMPO2**), shown in Fig. 1a and b.

We verified the bi-radical structure of **Ir-TEMPO2** by X-ray crystallography. The presence of a spin–spin interaction between the two TEMPO units in **Ir-TEMPO2** was investigated by EPR spectroscopy, luminescence spectroscopy and DFT calculations. The anticancer activity of these complexes was studied in a variety of cancer cell lines. The luminescence of the complexes allowed us to study their sub-cellular localisation using confocal microscopy.

## Results and discussion

### Synthesis and characterisation

The bipyridine ligands with nitroxide substituents (**L1** and **L2**) were synthesised by using amide-coupling reactions as shown in Schemes S1 and S2.† Complexes **Ir-TEMPO1** and **Ir-TEMPO2** were prepared by reacting **L1** and **L2** with the chloride-bridged phenylpyridine (N–C) cyclometallated iridium(iii) dimer [Ir(N–C)_2_(μ-Cl)]_2_. Detailed synthetic procedures are in the ESI (Schemes S3 and S4†). Ligands and complexes were characterised by ^1^H NMR, HRMS, and elemental analysis (details in the ESI†). The presence of free radicals in the ligands and complexes causes paramagnetic relaxation that leads to broadening of ^1^H NMR peaks. The ^1^H NMR resonances of **L2** and **Ir-TEMPO2** were much broader due to the presence of two TEMPO groups (Fig. S1†). Furthermore, the formation of **Ir-TEMPO2** was verified by single-crystal X-ray diffraction (Fig. 1e). The crystal structure determination showed that the iridium adopts octahedral geometry in **Ir-TEMPO2** with a distance between the two nitroxyl radicals of 8.63 Å.

### EPR studies

Continuous-wave EPR spectra of complexes **Ir-TEMPO1** and **Ir-TEMPO2** in solution are shown in Fig. 1c and d. In the case of the **Ir-TEMPO1**, hyperfine coupling to the ligand nitroxide ^14^N gives an equal-intensity three line spectrum, with a slight broadening of the high-field line, characteristic of a nitroxide radical undergoing tumbling in the fast motional regime.^23^,^24^ Similarly, three-line spectra were observed for the free ligands **L1** and **L2** (Fig. S3†). A much more complex spectrum was observed in the case of **Ir-TEMPO2**, with an additional six broad lines resolved. This pattern indicates the presence of an intramolecular electron–electron exchange interaction, *J*, which mixes the spin-states of the interacting radicals to generate singlet and triplet manifolds.^25^,^26^

In the limit of strong exchange (*J* ≫ *A*) where *A* is the hyperfine coupling, the system is well described by a total spin quantum number *S* = 1. This means that only transitions in the triplet manifold are observed and the spectrum is reduced to a simple 1 : 2 : 3 : 2 : 1 quintet. Similarly for vanishingly small exchange interaction (*J* ≪ *A*) the typical three-line spectrum of a non-interacting nitroxide radical is seen. In the intermediate exchange region, however, the state mixing induced by the hyperfine interaction makes *S* a poor quantum number, and a complicated spectral pattern emerges from the overlap of fifteen individual transitions, many of which are only partially allowed.^25^ The resonance fields are strongly dependent on the ratio of the exchange and hyperfine interactions, and hence spectral simulation permits the strength of the exchange interaction to be determined in the case of a rigid biradical.

The presence of both broad and narrow lines in the spectrum of **Ir-TEMPO2** is an indication that the biradical species is not rigid.^25^,^26^ Three of the transitions in the biradical are found at field positions that are independent of the magnitude of the exchange interaction, giving narrow lines at the field positions seen for a mono nitroxide species. As the field positions of the remaining transitions are dependent upon the exchange interaction, motional fluctuations tend to broaden the observed line shape. To produce a full spectral simulation is therefore non-trivial, requiring the motional dynamics to be considered. The interplay of variation in the electron exchange magnitude and rates of conformational dynamics is such that even in a full dynamic simulation, it is difficult to obtain a unique solution when fitting a single spectrum.^27^ Such an attempt was therefore not made, but as seen in Fig. 1d, reasonable spectral simulation was nevertheless obtained simply as a weighted sum of just two components having *J* = 0 and *J* = 43 MHz (*J*/*A* ≈ 1).^26^ It is however not possible to use this information to place firm bounds on the possible intermolecular approach distances between the two radicals. Simulation details are in the ESI (Fig. S5†).

Whereas an appreciable exchange interaction is observed in the case of **Ir-TEMPO2**, the typical three-line spectrum seen for **L2** (Fig. S3†) implies no significant exchange interaction. This is further supported by the observation of fine structure arising from partially resolved proton hyperfine couplings, showing that exchange broadening is absent in this system. These observations imply that metal binding must induce a conformational change in the ligand which permits closer approach of the nitroxide groups, assuming that the exchange interaction is dominated by through-space rather than through-bond coupling.

### Photophysical properties

**Fig. 2:**
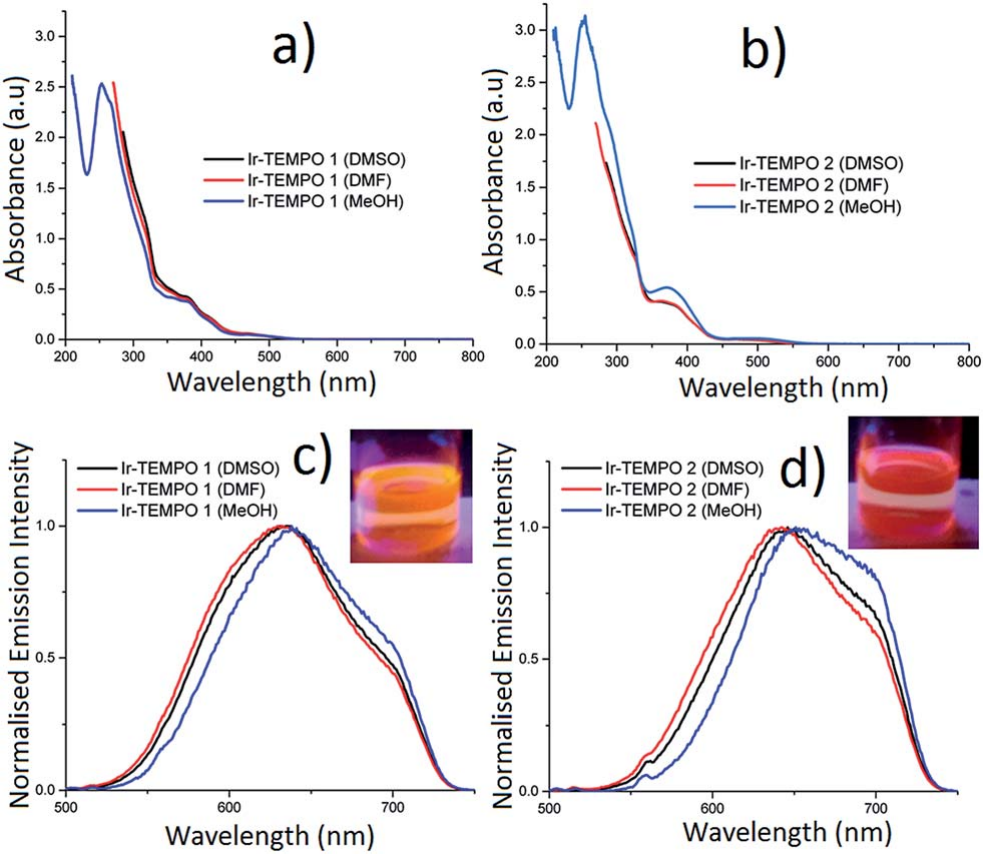
(a, b) UV-vis absorbance spectra; (c, d) uncorrected emission spectra of **Ir-TEMPO1** and **Ir-TEMPO2** in DMSO, DMF and methanol solutions (insets in c, d show the emission of **Ir-TEMPO1** and **Ir-TEMPO2** when excited with a 365 nm hand-held UV lamp in dichloromethane). The small feature around 560 nm in the emission spectra is due to Raman scattering from the solvent.

The absorption spectra of **Ir-TEMPO1** and **Ir-TEMPO2** were recorded in aerated DMSO, DMF and methanol and were similar in each solvent (Fig. 2). The strong absorption band around 260 nm (Fig. 2a and b) is assigned as a spin-allowed ligand-centred (^1^LC) transition, the absorbance at 380 nm is assigned as a ^1^MLCT transition and the weaker feature near 480 nm is assigned as a spin-forbidden ^3^MLCT. Both complexes show similar structured emission from 525 nm to beyond 700 nm in aerated DMSO, DMF and methanol solutions, following excitation at 480 nm (the extinction coefficient at 480 nm is *ca.* 1000 M^–1^ cm^–1^ for both samples in all three solvents). We attribute this broad emission to transitions from ^3^MLCT states, which would agree with previous studies of cyclometallated iridium(iii) complexes.^28^–^38^ For **Ir-TEMPO1**, the emission has a peak around 630 nm and there is a shoulder near 700 nm. The emission spectra for **Ir-TEMPO2** are similar but the peak is red-shifted by *ca.* 10 nm. This red shift in emission can be clearly distinguished visually with excitation at 365 nm using a hand held UV lamp (Fig. 2c and d inset). The emission quantum yields (Table 1) range from 1–5% and vary depending on the complex and the solvent; they are larger for **Ir-TEMPO1** and the solvent trend is DMSO > DMF > methanol. The emission detector's sensitivity drops off above 720 nm, causing an apparent cut off in the long-wavelength region (see ESI† for details). Therefore, the quantum yields that we report represent lower limits.

**Table 1:** Photophysical properties^*a*^ of **Ir-TEMPO1** and **Ir-TEMPO2** in DMSO, DMF and methanol

Compounds (solvent)	*τ*_1_ (μs)	*A*_1_ (%)	*τ*_2_ (μs)	*A*_2_ (%)	*χ* ^2^	*φ*_em_
**Ir-TEMPO1** (DMSO)	0.124	100	—	—	0.97	0.053 ± 0.0023
**Ir-TEMPO2** (DMSO)	0.155	5.45	0.067	94.6	0.98	0.025 ± 0.0013
**Ir-TEMPO1** (DMF)	0.096	100	—	—	1.02	0.037 ± 0.0010
**Ir-TEMPO2** (DMF)	0.099	33.4	0.062	66.6	1.00	0.022 ± 0.0005
**Ir-TEMPO1** (MeOH)	0.042	100	—	—	1.00	0.013 ± 0.0004
**Ir-TEMPO2** (MeOH)	0.047	12.0	0.033	88.0	0.96	0.008 ± 0.0004

^*a*^Luminescence lifetimes (*τ*_*i*_), their amplitudes (*A*_*i*_) and the *χ*^2^ goodness of fit parameter, and the emission quantum yields (*φ*_em_) ± standard deviation are shown. Excitation wavelengths were 480 nm (lifetime) and 470 nm (quantum yields); decays were recorded at the peak emission wavelength. Results from repeat decay measurements were in good agreement, and the uncertainties in reported values of lifetimes and amplitudes are ≤10%. Quantum yields are likely to be underestimated by *ca.* 10–20% (see ESI for details).

Luminescence lifetimes were determined for the same solutions using time-correlated single photon counting (TCSPC). When measured at the peak of the emission spectrum between 630–650 nm, **Ir-TEMPO1** displays a mono-exponential decay in the three different solvents (DMSO, DMF and methanol), while **Ir-TEMPO2** shows a bi-exponential decay in each solvent (Table 1). Both samples have long-lived excited-states with lifetimes ranging from 33–155 ns, typical of ^3^MLCT emission (Table 1). Heterogeneity in the photophysics of organic molecules is usually attributed to a variation in the molecule's local environment, conformational change, protonation or other well-defined structural changes. In contrast, heterogeneous decays and spectra for a range of organometallic complexes have often been ascribed to dual emission *e.g.* from two localised MLCT states.^39^ As discussed in a recent comprehensive review,^40^ dual emission appears to be a feature of the photophysics of most organometallic complexes, albeit not always in solution under ambient conditions. Furthermore, the “dual” emission usually appears to be due to more than two states or a continuum of states.^40^ The authors of the review discuss in detail potential explanations for these findings, particularly in the context of charge transfer states and ion pairing. It is also clear, however, that there are complexes that show no or little evidence of dual emission.^40^ The dual emission is most common for mixed-ligand complexes with asymmetric ligands. Both complexes reported here are mixed-ligand species with two ppy ligands and a bipy with one or two TEMPO units attached. Interestingly, we find that **Ir-TEMPO1**, which has the asymmetrical **L1** ligand displays a single-exponential decay, while **Ir-TEMPO2**, where the **L2** ligand is symmetrical, has a clear double exponential decay. To investigate further the origin of the two lifetimes, we performed a global analysis where we recorded the decays of **Ir-TEMPO2** as a function of both excitation and emission wavelength. By exciting at 450, 480 and 510 nm and detecting at 580, 640 and 700 nm, we collected a total of 9 decays (Table S1†). The global analysis involved fitting each decay with the same lifetimes, but allowing the weights and values of the lifetimes to vary during the fitting. Such an analysis is an excellent method for revealing heterogeneity that is not apparent for an individual decay. In this case, however, we found that the data can indeed be explained by only two unique lifetimes. The global fits with two lifetimes are excellent with a global *χ*^2^ of 0.98 in contrast a global fit with a single lifetime gave a value of 6.0. In light of these data and the preceding discussion, we attribute the bi-exponential decay of **Ir-TEMPO2** to the presence of two distinct conformations. The two conformations are assigned as ground-state species because the relative weights of the two decay components, at a particular emission wavelength, changes with excitation wavelength; these weights should be independent of excitation wavelength for an excited-state reaction. Furthermore, a rise time is often seen at longer emission wavelengths for excited state reactions, but none was observed here. Finally, we note that the minor decay component for **Ir-TEMPO2** has a lifetime that is very similar to that of **Ir-TEMPO1** in the same solvent, suggesting that this results from a conformation in which the TEMPO units in ligand **L2** are independent of each other.

### Computational study of **Ir-TEMPO2**

**Fig. 3:**
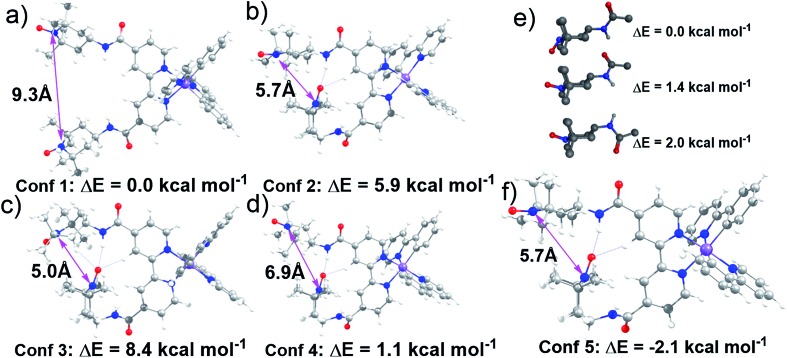
(a–d) Structures of selected conformations of **Ir-TEMPO2** optimised at the BP/ZORA/SVP/D3/COSMO level showing the nitroxyl–nitroxyl N···N distance; (e) three lowest-energy conformers from low-mode MD conformational search for TEMPO-amide. Non-polar hydrogen atoms are omitted for clarity; (f) DFT-optimised structure for lowest-energy **Ir-TEMPO2** complex. The energies were calculated for these structures using a larger, more accurate, basis set (TZVP).

Lifetime measurements and EPR studies of **Ir-TEMPO2** suggested the presence of more than one low-energy conformation. In order to explore the other possible conformations, an *ad hoc* ligand field molecular mechanics (LFMM)^41^ force field (FF) was constructed to facilitate large stochastic searches. Favourable conformations were then subjected to further DFT analysis. The selection of ‘favourable’ conformations was based on the X-ray conformation as conformation **1** and then three more conformations chosen from the LFMM results. Conformations were selected within 7 kcal mol^–1^ of the best LFMM energy with an N···N distance <6 Å and a noticeably different conformation compared to the best from LFMM. Table S6† displays the initial DFT results. The larger, more accurate basis sets suggest that the X-ray-like conformation leads to the lowest-energy structure although conformation **4** is within a few kcal mol^–1^. The structures of all four conformations at the BP/ZORA/SVP/D3/COSMO level are shown in Fig. 3 and the Cartesian coordinates are in the ESI.†

The presence of H-bonding between one of the TEMPO groups and the neighbouring amide leads to a significantly shorter distance between the two nitroxyl radicals. The N···N distance drops from *ca.* 9 Å to 7 Å from conformation **1** to conformation **4**. However, we could not rule out the possibility that other conformations with low energies and different N···N distances exist. To partially address this issue, a low-mode molecular dynamics (MD) conformational search of just an amido-TEMPO unit was undertaken to assess whether there might be any other low-energy conformations available to **Ir-TEMPO2**. The three lowest-energy conformers are shown in Fig. 3e and the relative energies were confirmed with DFT calculations optimised at the B3LYP/SVP/COSMO (CH_2_Cl_2_) level with TZVP single-point energies. Both molecular mechanics (MM) and DFT suggest that rotation of the TEMPO group relative to the amide moiety might generate an energetically-accessible conformation for the **Ir-TEMPO2**. Inspection of Fig. 3 indicates that an approximately 180° rotation of the ‘terminal’ TEMPO unit would generate a shorter N···N contact only for conformation **4** (Fig. 3d).

The DFT-optimised structure is shown in Fig. 3f and the Cartesian coordinates are in the ESI.† Not only does conformation **5** have a significantly shorter nitroxyl–nitroxyl N···N distance of only 5.7 Å, but it is also 2.1 kcal mol^–1^ more stable than conformation **1** at the BP/ZORA/TZVP/D3/COSMO (CH_2_Cl_2_) level. The computational results indicate that there is more than one accessible conformation for **Ir-TEMPO2** and that these conformers are likely to have significantly different N···N separations. The EPR signal could thus comprise a component from a conformer with a large N···N separation which yields a ‘normal’ three-line nitroxyl signal together with one or perhaps more components where the N···N distance is significantly shorter leading to coupling between the two nitroxyl units.

### *In vitro* antiproliferative activity

The antiproliferative activity of **Ir-TEMPO1**, **Ir-TEMPO2**, and cisplatin (CDDP) against A2780 human ovarian, cisplatin-resistant A2780Cis human ovarian, A549 human lung and PC3 human prostate cancer cells was determined. Their selectivity was also investigated by comparing their toxicity in cancer cells with that towards the non-cancerous human lung fibroblast cell line (MRC5). The IC_50_ values of **Ir-TEMPO2** are *ca.* 2–30× lower than for **Ir-TEMPO1** in all the cell lines tested, suggesting a significant role for the TEMPO radical unit in anticancer activity. **Ir-TEMPO2** is 5× more active than cisplatin against cisplatin-resistant A2780Cis ovarian cancer cells, showing no cross-resistance with the platinum drugs and suggesting a different mechanism of action. For comparison we also determined the antiproliferative activity of the precursors [Ir(N–C)_2_(μ-Cl)]_2_, and [Ir(ppy)_2_(bpy)]^+^, as analogues without the TEMPO units, and mono- and bis-TEMPO ligands **L1** and **L2**. The dimer and [Ir(ppy)_2_(bpy)]^+^ were inactive up to the concentrations tested (100 μM), and the ligands were only weakly active (IC_50_ values 74–84 μM, Table S7†). Importantly, the complexes **Ir-TEMPO1** and **Ir-TEMPO2** are more potent in all cell lines than related free ligands. Furthermore, both complexes were less toxic towards MRC5 normal lung fibroblasts (Table 2). **Ir-TEMPO2** is highly active towards the PC3 prostate cancer cell line with an IC_50_ value of 0.53 μM, 8× more potent than the clinical drug cisplatin (CDDP), and importantly *ca.* 15-fold selective for this cancer cell line compared with non-cancerous MRC5 cell line (Tables 2 and S7†).

**Table 2:** IC_50_ values for **Ir-TEMPO1**, **Ir-TEMPO2**, and cisplatin (CDDP) against A2780 human ovarian, A2780Cis cisplatin-resistant human ovarian, A549 human lung, PC3 human prostate cancer cell lines, and normal MRC5 human lung fibroblast cell line. Corresponding values for precursors can be found in the ESI (Table S7)

Cell lines	IC_50_ (μM)		
	**Ir-TEMPO1**	**Ir-TEMPO2**	CDDP
A2780	14.5 ± 0.5	3.0 ± 0.2	1.2 ± 0.2
A2780Cis	8.6 ± 0.1	2.57 ± 0.08	13.4 ± 0.3
A549	13.8 ± 0.5	7.5 ± 0.6	3.2 ± 0.1
PC3	16.21 ± 0.08	0.53 ± 0.02	4.1 ± 0.5
MRC5	32.7 ± 0.5	8.2 ± 0.7	12.8 ± 0.4

### Antioxidant activity

**Fig. 4:**
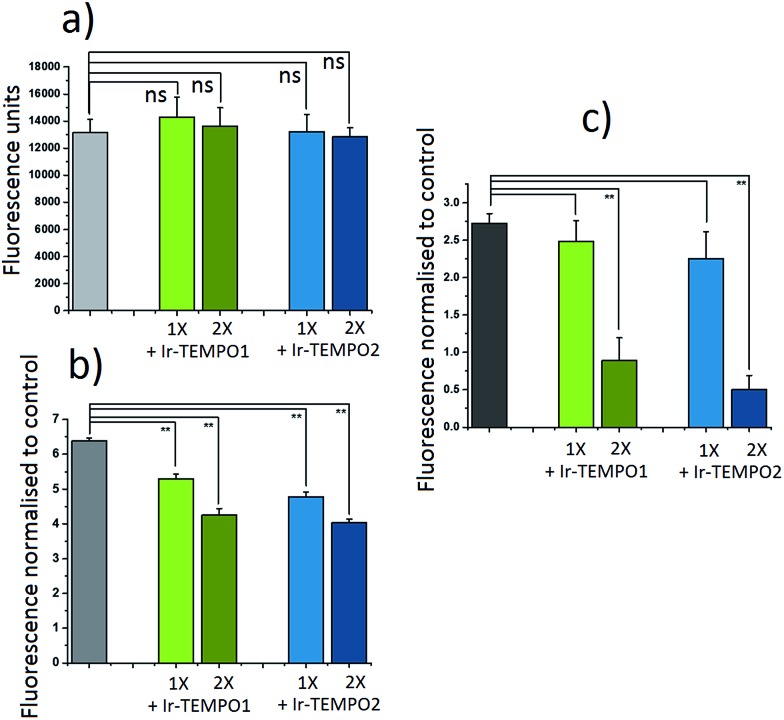
Antioxidant activity of **Ir-TEMPO1** and **Ir-TEMPO2** in A2780 ovarian cancer cells. (a) Comparison between the fluorescence of 2′,7′-dichlorofluorescein diacetate (DCFH-DA) on its own or in the presence of equipotent concentrations of **Ir-TEMPO1** or **Ir-TEMPO2**. (b, c) The relative fluorescence normalised to the controls of A2780 cancer cells stained with DCFH-DA when ROS has been induced with hydrogen peroxide (575 μM, b) or *tert*-butyl hydroperoxide (TBHP) (250 μM, c). In all cases: the concentrations of the complexes used were equipotent with 1- or 2-fold of their IC_50_ concentrations in this cancer cell line, drug exposure to the iridium complexes was 24 h prior to staining, followed by 4 h of ROS induction. Fluorescence measurements were carried out using excitation at 485 nm and emission at 530 nm for DCFH-DA. Each measurement was carried out in duplicates of triplicates and their statistical significance was determined using an independent two-sample *t*-tests with unequal variances, Welch's tests, (*p* < 0.01 for **, and *p* < 0.05 for *).

The antioxidant activity of complexes **Ir-TEMPO1** and **Ir-TEMPO2** was determined in A2780 ovarian cancer cells using 2′,7′-dichlorofluorescein diacetate (DCFH-DA) and ROS induction by hydrogen peroxide as well as the organic hydroperoxide TBHP (*tert*-butyl hydroperoxide), as shown in Fig. 4. The fluorescence based-experiment confirmed that, as expected, **Ir-TEMPO1** and **Ir-TEMPO2** do not induce intracellular ROS *per se*, as there is no increased DCFH-DA fluorescence upon drug exposure. Furthermore, it also shows a reduction in fluorescence when cells have been pre-treated with **Ir-TEMPO1** and **Ir-TEMPO2** for 24 h prior to induction of ROS by hydrogen peroxide or TBHP. This reduction of fluorescence is concentration-dependent, as the values for cells treated with equipotent IC_50_ concentrations of the TEMPO-appended complexes are higher than those treated with 2-fold the IC_50_ values (Fig. 4). Consistent with the antiproliferative activity results, complex **Ir-TEMPO2** shows higher antioxidant activity than **Ir-TEMPO1**, showing again the influence of the TEMPO radical in these complexes.

### Cell imaging studies

**Fig. 5:**
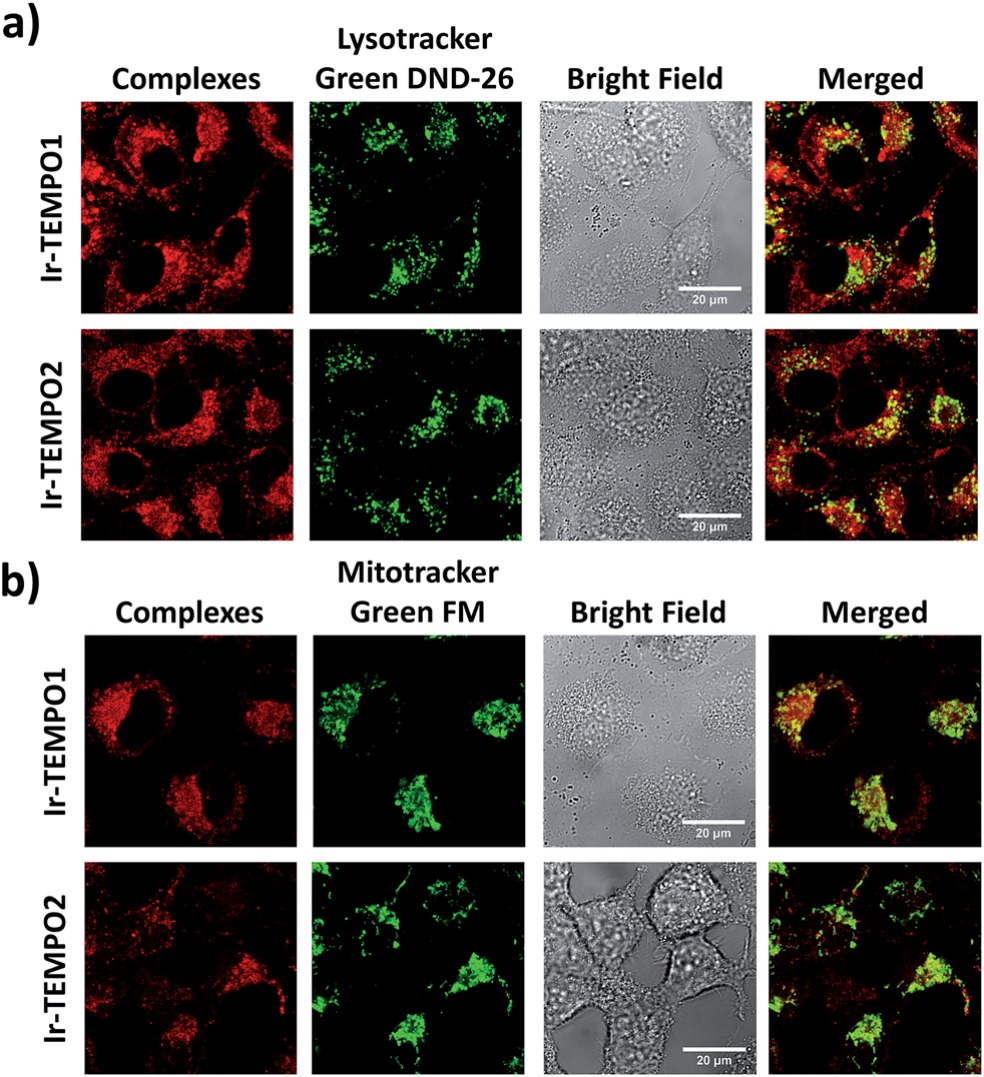
Confocal microscope images of **Ir-TEMPO1** and **Ir-TEMPO2** in PC3 prostate adenocarcinoma cells. (a) Co-localisation with MitoTracker Green FM (400 nM; 30 min at 37 °C; *λ*_ex_ = 490 nm/*λ*_em_ = 516 nm). (b) Co-localisation with LysoTracker Green DND-26 (50 nM; 30 min at 37 °C; *λ*_ex_ = 504 nm/*λ*_em_ = 511 nm).

The strong luminescence of **Ir-TEMPO1** and **Ir-TEMPO2** allowed us to study their sub-cellular targets in PC3 prostate adenocarcinoma cells by optical imaging. The cells were seeded in 8-well microscopy chambers and left to attach for 48 h, and then treated for another 4 h with equipotent IC_50_ concentrations of **Ir-TEMPO1** (16 μM) and **Ir-TEMPO2** (0.5 μM). Cells were then washed with phosphate buffer saline (PBS) and fresh phenol-red-free medium to remove the excess complexes that were not taken up by cells. Confocal microscopy showed that **Ir-TEMPO1** and **Ir-TEMPO2** were taken up by PC3 prostate adenocarcinoma cells (*λ*_ex_ = 405 nm/*λ*_em_ = 600 nm). Co-localisation experiments of **Ir-TEMPO1** and **Ir-TEMPO2** using LysoTracker Green DND-26 and MitoTracker Green FM revealed that both complexes were highly localised in mitochondria, with only very minimal localisation in lysosomes (Fig. 5). Control experiments with and without complexes, LysoTracker Green and MitoTracker Green confirmed that the emission is observed only in the presence of complexes (Fig. S6†).

### Mitochondrial membrane potentials

**Fig. 6:**
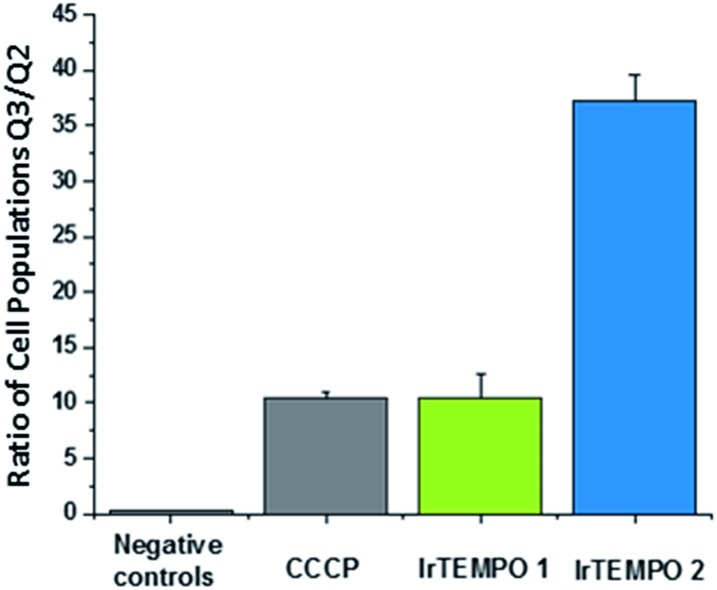
Ratio of cellular populations in Q3/Q2 of a flow cytometry dot plot for PC3 cells exposed for 24 h to **Ir-TEMPO1** and **Ir-TEMPO2** and treated with JC10 mitochondrial staining.

After confirming by optical imaging that complexes **Ir-TEMPO1** and **Ir-TEMPO2** localise highly in mitochondria, we investigated their effects on the mitochondrial membrane potential. For this, we carried out flow cytometry analysis of PC3 cells exposed to equipotent concentrations of the complexes stained with JC-10. This dye accumulates selectively in cellular mitochondria as red fluorescent aggregates. Upon mitochondrial membrane potential changes, it leaks as a monomeric green fluorescent form. This experiment included cells treated with carbonyl cyanide *m*-chlorophenyl hydrazone (CCCP) as positive controls and untreated cells as negative controls, both of which were also used for compensation purposes. In both cases, **Ir-TEMPO1** and **Ir-TEMPO2** induced depolarisation of the mitochondrial membrane. Flow cytometry dot plots establish four different cellular populations: Q1, high red fluorescence, Q2 high red and green fluorescence, Q3 high green fluorescence and Q4 low fluorescence (Fig. S7†). The ratio between the cellular populations in Q3 and Q2 indicates cellular populations in which the JC-10 dye has leaked from the mitochondria and has generated high green fluorescence as a response to changes in the membrane potential. **Ir-TEMPO1** induces similar changes to the positive control CCCP (ratios of 10.4 and 10.5 respectively) while **Ir-TEMPO2** is capable of achieving a Q3/Q2 ratio of 37.2 (Fig. 6).

## Conclusions

We have designed octahedral cyclometallated iridium(iii) complexes containing one and two TEMPO nitroxide spin labels. They are highly luminescent with long emission lifetimes (33–155 ns). In the complex **Ir-TEMPO2**, unlike in the free bisTEMPO ligand (**L2**), there is a communication between the two nitroxide radicals. The EPR spectra (Fig. 1d) not only showed evidence for strong spin–spin exchange interaction between the nitroxide radicals, but also for their conformational flexibility. Fluorescence lifetime measurements also showed the existence of more than one energetically accessible conformation for **Ir-TEMPO2**. A second conformation (in addition to that seen in the X-ray structure) with 2.1 kcal mol^–1^ lower energy was revealed by DFT calculations and involved in hydrogen bonding between the amide of one TEMPO unit and that of a neighbouring TEMPO (Fig. 3).

The anticancer activity of the bis-nitroxide complex **Ir-TEMPO2** is 2× (A549 lung cancer cells) to 15× (PC3 prostate cancer cells) higher compared to the one TEMPO containing complex **Ir-TEMPO1** (Table 2) showing an important role for the TEMPO radical in the antiproliferative activity. Moreover, these complexes are not cross-resistant with cisplatin in ovarian cancer cells, and **Ir-TEMPO2** is 7× more active than CDDP against PC3 prostate cancer cells and 15× more selective *versus* normal cells (MRC5 fibroblast cells). The antioxidant activity of **Ir-TEMPO1** and **Ir-TEMPO2** was demonstrated in ovarian cancer cells. Luminescence images showed that the complexes localise in mitochondria of PC3 prostate cancer cells and flow cytometry studies have confirmed that both iridium(iii) complexes induce changes in the mitochondria membrane potential of cancer cells. Mitochondria are attractive as target sites for anticancer drugs because they are known to malfunction in cancer cells.^42^

## Conflicts of interest

There are no conflicts to declare.

## Supplementary Material

Supplementary information

Crystal structure data

## References

[cit1] Holmes D. (2015). Nature.

[cit2] Liu Z., Canelon I. R., Qamar B., Hearn J. M., Habtemariam A., Barry N. P., Pizarro A. M., Clarkson G. J., Sadler P. J. (2014). Angew. Chem., Int. Ed..

[cit3] Liu Z., Habtemariam A., Pizarro A. M., Fletcher S. A., Kisova A., Vrana O., Salassa L., Bruijnincx P. C. A., Clarkson G. J., Brabec V., Sadler P. J. (2011). J. Med. Chem..

[cit4] Novohradsky V., Zerzankova L., Stepankova J., Kisova A., Kostrhunova H., Liu Z., Sadler P. J., Kasparkova J., Brabec V. (2014). Metallomics.

[cit5] Liu Z., Canelon I. R., Habtemariam A., Clarkson G. J., Sadler P. J. (2014). Organometallics.

[cit6] Wilbuer A., Vlecken D. H., Schmitz D. J., Kraling K., Harms K., Bagowski C. P., Meggers E. (2010). Angew. Chem., Int. Ed..

[cit7] Cao J. J., Tan C. P., Chen M. H., Wu N., Yao D. Y., Liu X. G., Jia L. N., Mao Z. W. (2016). Chem. Sci..

[cit8] Ye R. R., Tan C. P., Ji L. N., Mao Z. W. (2016). Dalton Trans..

[cit9] He L., Li Y., Tan C. P., Ye R. R., Chen M. H., Cao J. J., Ji L. N., Mao Z. W. (2015). Chem. Sci..

[cit10] Hubbell W. L., Gross A., Langen R., Lietzow M. A. (1998). Curr. Opin. Struct. Biol..

[cit11] Soule B. P., Hyodo F., Matsumoto K., Simone N. L., Cook J. A., Krishna M. C., Mitchell J. B. (2007). Free Radical Biol. Med..

[cit12] Gariboldi M. B., Lucchi S., Caserini C., Supino R., Oliva C., Monti E. (1998). Free Radical Biol. Med..

[cit13] Suy S., Mitchell J. B., Ehleiter D., Friedman A. H., Kasid U. (1998). J. Biol. Chem..

[cit14] Arion V. B., Dobrov A., Goschl S., Jakupec M. A., Keppler B. K., Rapta P. (2012). Chem. Commun..

[cit15] Dobrova A., Göschl S., Jakupec M. A., Bijelić A. P., Gräslund A., Rapta P., Arion V. B. (2013). Chem. Commun..

[cit16] Sen' V. D., Golubev V. A., Volkova L. M., Konovalova N. P. (1996). J. Inorg. Biochem..

[cit17] Sen' V. D., Terent'ev A. A., Konovalova N. P. (2011). Russ. Chem. Bull..

[cit18] Sen' V. D., Tkachev V. V., Volkova L. M., Goncharova S. A., Raevskaya T. A., Konovalova N. P. (2003). Russ. Chem. Bull..

[cit19] Venkatesh V., Wedge C. J., Canelón I. R., Habtemariam A., Sadler P. J. (2016). Dalton Trans..

[cit20] Yang J., Cao Q., Hu W. L., Ye R. R., He L., Ji L. N., Qin P. Z., Mao Z. W. (2017). Dalton Trans..

[cit21] Xu Y., Kalyanaraman B. (2007). Free Radical Res..

[cit22] Fulda S., Galluzzi L., Kroemer G. (2010). Nat. Rev. Drug Discovery.

[cit23] Stone T. J., Buckman T., Nordio P. L., McConnell H. M. (1965). Proc. Natl. Acad. Sci. U. S. A..

[cit24] Chechik V., Wellsted H. J., Korte A., Gilbert B. C., Caldararu H., Ionita P., Caragheorgheopol A. (2004). Faraday Discuss..

[cit25] Bencini A. and Gatteschi D., EPR of exchange coupled systems, Dover, New York, 2012, (Original work published 1990, Berlin: Springer-Verlag).

[cit26] Luckhurst G. R., Pedulli G. F. (1970). Mol. Phys..

[cit27] Parmon V. N., Zhidomirov G. M. (1974). Mol. Phys..

[cit28] Howarth A. J., Davies D. L., Lelj F., Wolf M. O., Patrick B. O. (2014). Inorg. Chem..

[cit29] Maggioni D., Galli M., D'Alfonso L., Inverso D., Dozzi M. V., Sironi L., Iannacone M., Collini M., Ferruti P., Ranucci E., D'Alfonso G. (2015). Inorg. Chem..

[cit30] Yi S., Kim J. H., Cho Y. J., Lee J., Choi T. S., Cho D. W., Pac C., Han W. S., Son H. J., Kang S. O. (2016). Inorg. Chem..

[cit31] Law W. H. T., Lee L. C. C., Louie M. W., Liu H. W., Ang T. W. H., Lo K. K. W. (2013). Inorg. Chem..

[cit32] Li S. P. Y., Liu H. W., Zhang K. Y., Lo K. K. W. (2010). Chem.–Eur. J..

[cit33] Liu H. W., Zhang K. Y., Law W. H. T., Lo K. K. W. (2010). Organometallics.

[cit34] Law W. H.-T., Leung K.-K., Lee L. C.-C., Poon C. S., Liu H. W., Lo K. K. W. (2014). ChemMedChem.

[cit35] Lo K. K. W., Chung C. K., Lee T. K. M., Lui L. H., Tsang K. H. K., Zhu N. (2003). Inorg. Chem..

[cit36] Lo K. K. W., Ng D. C. M., Chung C. K. (2001). Organometallics.

[cit37] Lo K. K. W., Chung C. K., Ng D. C. M., Zhu N. (2002). New J. Chem..

[cit38] Zhang K. Y., Liu H. W., Tang M. C., Choi A. W., Zhu N., Wei X. G., Lau K. C., Lo K. K. W. (2015). Inorg. Chem..

[cit39] Glazer E. C., Magde D., Tor Y. (2005). J. Am. Chem. Soc..

[cit40] Magde D., Magde M. D., Glazer E. C. (2016). Coord. Chem. Rev..

[cit41] Deeth R. J. (2001). Coord. Chem. Rev..

[cit42] Yang Y., Karakhanova S., Hartwig W., Dhaese J. G., Philippov P. P., Werner J., Bazhin A. V. (2016). J. Cell. Physiol..

