# Supplementary material for: Mitochondria-targeted spin-labelled luminescent iridium anticancer complexes
Source: Chem Sci. 2017 Oct 20;8(12):8271–8. doi: 10.1039/c7sc03216a (PMC5857930; doi:10.1039/c7sc03216a)
Supplement: Supplementary file 1 [file SC-008-C7SC03216A-s001.pdf]

Electronic Supplementary Material (ESI) for Chemical Science.
This journal is © The Royal Society of Chemistry 2017

Supporting Information

Mitochondria-Targeted Spin-labelled Luminescent Iridium Anticancer Complexes

V. Venkatesh, Raul Berrocal-Martin, Christopher J. Wedge, Isolda Romero-Canelón, Carlos Sanchez-Cano, Ji-Inn Song, James P. C. Coverdale, Pingyu Zhang, Guy Clarkson, Abraha Habtemariam, Steven W. Magennis, Robert J. Deeth and Peter J. Sadler*

Materials and methods

X-ray crystal structure

EPR instrumental and simulation details

Steady-state UV-visible and luminescence spectroscopy

Luminescence lifetime measurements using time-correlated single-photon counting (TCSPC)

Photoluminescence quantum yield

Cell culture

In vitro growth inhibition assay

Antioxidant activity

Mitochondrial membrane potential assay

Cell imaging

Computational studies

Synthetic procedures (Schemes S1-S4)

Synthesis of ligand **L1**

Synthesis of ligand **L2**

Synthesis of [Ir (N-C)$_2$ L1] PF_6(**Ir-TEMPO 1**)

Synthesis of [Ir(N-C)$_2$ L2] PF_6(**Ir-TEMPO 2**)

Tables S1-S8

Figures S1-S7

References

Materials and methods

4-Amino-2,2,6,6-tetramethylpiperidine-1-oxyl, N-(3-dimethylaminopropyl)-N′-ethylcarbodiimide-hydrochloride, N,N-diisopropylethylamine, N,N-dimethylformamide, dimethyl sulfoxide-d_6 99.5 atom %D, and 2,2′-bipyridine-4,4′-dicarboxylic acid were purchased from Sigma-Aldrich. 4'-Methyl-2,2'-bipyridine-4-carboxylic acid was obtained from Carbosynth Limited, UK and N-hydroxysuccinimide and $AgPF_6$ from Aldrich. Dichloromethane and methanol were purchased from Fisher Scientific at laboratory reagent grade.

ESI-HR-MS spectra were recorded on a Bruker microTOF instrument. ^1H NMR spectra were obtained on a 400 MHz Bruker Avance III HD spectrometer.

X-ray crystal structure

Single crystals of $C_{52}H_{58}F_6Ir_1N_8O_4P_1$ (**Ir-TEMPO2**) were grown from methanol. A suitable crystal was selected and mounted on a Mitegen head with Fromblin oil and placed on an Xcalibur Gemini diffractometer with a Ruby CCD area detector. The crystal was kept at 150(2) K during data collection. Using Olex2,[1] the structure was solved with the ShelXS[2] structure solution program using Patterson Method and refined with the ShelXL[3] refinement package using Least Squares minimisation. The data showed that **Ir-TEMPO2** crystals belong to the orthorhombic space group $Cmc2_1$. The asymmetric unit contains half an **Ir-TEMPO2** molecule. The Ir atom sits on a mirror plane which bisects the substituted bipyridyl ligand. Both the ligands and the PF_6 were disordered over this mirror plane. The Cambridge Crystallographic Data Centre deposition number is CCDC 1522104. A copy of this information can be obtained free of charge on application to CCDC, 12 Union Road, Cambridge CB21EZ, UK. [Fax: +44-1223/336-033; E-mail: deposit@ccdc.cam.ac.uk].

EPR Instrumental and simulation details

EPR measurements were performed at X-band (~9.87 GHz) on a Bruker EMX spectrometer using a cylindrical cavity (Bruker ER41022SHQE). Samples were prepared in dichloromethane at a concentration of 0.1 mM, and due to dielectric loss of the solvent were contained within 2.2 mm inner diameter quartz tubes (Wilmad 705-SQ). Dissolved oxygen was removed by a minimum of three freeze-pump-thaw cycles, and tubes were subsequently backfilled with dry nitrogen. Observation of fine structure arising from ^1H hyperfine couplings at the low modulation depth used (10 µT at 100 kHz) confirms efficient removal of oxygen which would otherwise broaden the EPR lines. Measurements were performed with 4096 points recorded across an 8 mT sweep width, using a conversion time of 20.48 ms and time constant of 10.24 ms. The choice of microwave power of ~3.2 mW (microwave attenuation 18 dB) was based on microwave power saturation experiments which confirmed operation within the linear regime. No significant differences were observed between the power saturation characteristics of any of the samples studied, Figure S4.

All EPR spectral simulations were carried out in Matlab using the EasySpin package.[4] For the mono-nitroxide complex, **Ir-TEMPO1** the simulations were performed using the *garlic* routine which is applicable to solution spectra in the fast motional regime. Using parameters for a similar kind of nitroxide complex as our starting point,[5] the isotropic g-value and ^{14}N hyperfine constant were first scaled manually to obtain a reasonable overall fit. Hybrid methods in the *esfit* routine were then applied using both genetic and Levenberg/Marquardt algorithms to determine values for the additional ^{1}H hyperfine couplings, which were treated as three pairs of equivalent protons. The final EasySpin system structure used was as follows (linewidths are in units of mT and hyperfine couplings in units of MHz):

```
Sys =
          S:  0.5000
          G:  [2.0125 2.0122 2.0070]
       Nucs:  'N,(12,13)C,1H,1H,1H,1H,1H,1H '
         A_:   44.47      19.277    0          14N
               17.9269    0         0          13C
               2.1403     0         0          1Ha
               2.1403     0         0          1Ha
               1.4980     0         0          1Hb
               1.4980     0         0          1Hb
               0.9841     0         0          1Hc
               0.9841     0         0          1Hc
      Abund:  {[1]  [0.9786 0.0214]  [1]  [1]  [1]  [1]  [1]  [1]}
         lw:  [0 0.0507]
  logtcorr:   −10.0391                         i.e. τc = 91 ps
```

Steady-state UV-visible and luminescence spectroscopy

Solvents used were DMSO (spectrophotometric grade, Sigma-Aldrich), DMF (spectrophotometric grade, Alfa Aesar) and methanol (spectrophotometric grade, Sigma-Aldrich). Diluted samples were prepared for spectroscopy measurements. Solutions were air-saturated. Solvents had negligible fluorescence under the conditions used.

UV-vis spectra were measured using an Agilent Cary 60 Spectrophotometer (Agilent Technologies, UK), at wavelengths from 200 nm to 800 nm with 0.5 nm intervals. Absorbance at 480 nm was less than 0.1 in order to avoid inner filter effects. Extinction coefficients at 480 nm were determined for each sample in the different solvents. Emission spectra (with 480 nm excitation) were measured using xenon arc lamp excitation with a FluoTime 300 spectrometer (Picoquant, Germany) equipped with a hybrid PMT detector (PMA Hybrid 40, Picoquant) by scanning the emission wavelength from 500 nm to 750 nm with an interval of 1 nm.

Luminescence lifetime measurements using time-correlated single-photon counting (TCSPC)

Time-resolved fluorescence spectroscopy was performed using the technique of TCSPC with the FluoTime 300 spectrometer. Solution conditions were as described above for steady-state experiments. The excitation source was a super continuum laser (WL-SC-400-4-PP, Fianium) with tuneable wavelength filter and multimode fibre (Superchrome-Vis-FDS-MM, Fianium) at a wavelength of 480 nm, bandpass of 10 nm and at a repetition rate of 0.20 MHz. The instrument response function (IRF) was *ca.* 130 ps full width at half maximum; the time/channel was 128 ps. Magic-angle conditions were used. For global analysis, samples were excited and detected at three different wavelengths and the nine decays were fitted simultaneously with lifetimes as common parameters. Decay curves were analysed with tail fitting from the peak of the decay (FluoFit, Picoquant GmbH). The quality of fit was judged on the basis of the reduced chi-squared statistic, χ^2, and the randomness of residuals.

Photoluminescence quantum yield

Quantum yields for **Ir-TEMPO1** and **Ir-TEMPO2** samples in three different solvents were determined using the relative method. Corrected emission spectra were obtained, under magic-angle conditions, using the FluoTime 300 spectrometer and Fianium laser described above (exc. 470 nm, 10 nm bandpass, 10 MHz rep. rate). Fluorescein in 0.1M NaOH, with a reported quantum yield of 0.92,[6] was used as reference fluorophore. The spectral sensitivity of the detector on the FluoTime 300 spectrometer drops off steeply above *ca.* 720 nm (causing the noticeable cut-off observed in the emission spectra). Therefore, the range used to calculate the area of the emission spectra was 480–730nm. We estimate that the actual quantum yields are *ca.*10-20% higher, assuming a regular emission spectral profile.

Cell culture

Human ovarian carcinoma cell line (A2780), its cisplatin-resistant derived cell line (A2780Cis), human lung carcinoma cell line (A549), human prostate cancer cell line (PC3) and non-cancerous human lung fibroblast cell line (MRC5) were obtained from the European Collection of Cell Cultures (ECACC). All cells were used between passages 5 to 18 and were grown in Roswell Park Memorial Institute medium (RPMI-1640) supplemented with foetal calf serum (10% v/v), 2 mM glutamine (1% v/v) and penicillin/streptomycin (1% v/v). In all cases, cells were grown as adherent monolayers at 310 K in a 5% CO_2 humidified atmosphere and passaged at ca. 70-80% confluence.

In vitro growth inhibition assay

Approximately, 5000 cells were seeded per well in 96-well plates. The cells were pre-incubated in drug-free media at 310 K for 48 h before adding different concentrations of the compounds (**Ir-TEMPO1** and **Ir-TEMPO2**) to be tested. Stock solutions of **Ir-TEMPO1** and **Ir-TEMPO2** were firstly prepared in 5% DMF (v/v) and a mixture 0.9% saline and medium (1:1)

(v/v) following serial dilutions in RPMI-1640. The drug exposure period was 24 h. After this, supernatants were removed by suction and each well was washed with PBS (phosphate-buffered saline). Then the cells were allowed to recover for 72 h in a drug-free medium at 310 K. Sulforhodamine B colorimetric assay was used to determine cell viability. The absorbance values of the solubilised dye allowed the determination of viable treated cells compared to untreated controls. IC_{50} values (concentrations which caused 50% of cell death), were determined as duplicates of triplicates in two independent sets of experiments and their standard deviations were calculated. The concentrations of Iridium (λ = 208.882, 237.277) in the biological stock solutions of **Ir-TEMPO1** and **Ir-TEMPO2** were determined by ICP-OES using a Perkin Elmer Optima 5300 DV Optical Emission Spectrophotometer. Calibration curve of Iridium (50-700 ppb) was freshly prepared in nitric acid (3.6% v/v) with standard addition of sodium chloride to match the sample matrix. Total dissolved solids (TDS) did not exceed 0.2 % w/v.

Antioxidant activity

A2780 ovarian carcinoma cells were seeded in 96-well black plates using 10,000 cells per well. Cells were allowed to attach for 24 h before adding 1- or 2-fold equipotent concentrations of **Ir-TEMPO1** or **Ir-TEMPO2** equivalent to their IC_{50} values in this cell line (14 and 3 µM, respectively). After 24 h of drug exposure, supernatants were removed by suction and the plates were washed with PBS. To each well, 100 µL of a 50 µM solution of 2',7'–dichlorofluorescein diacetate(DCFH-DA) were added and the plates were incubated with the dye in the dark for 1 h at 310 K. Once cells were stained, supernatants were removed by suction and wells were washed with PBS before adding the ROS inducers. Hydrogen peroxide was used at 0.575 mM and tert-butyl hydroperoxide (TBHP) at 250 µM. ROS induction was allowed for 4 h in the dark at 310 K. Fluorescence readings were obtained with excitation at 485 nm and emission at 530 nm. This experiment included negative untreated controls, controls only treated with the iridium complexes (to discard auto-fluorescence), untreated cells with hydrogen peroxide or TBHP, and complex treated cells with the ROS inducers. The two last controls were carried out for background subtraction.

Mitochondrial membrane potential studies

Human prostate cancer cells PC3 were seeded in a 6-well plate using 1.0 x 10^6 cells/well. They were allowed to attach in drug-free medium for 24 h at 310 K in a 5% CO_2 humidified atmosphere before being treated for further 24 h with **Ir-TEMPO1** and **Ir-TEMPO2** at equipotent concentrations equal to their IC_{50} in the corresponding cell line. After this time, supernatants were removed by suction, cells were washed with PBS and cell pellets were collected after using trypsin. Washed cell pellets were stained in the dark using the JC-10 Mitochondrial membrane potential kit according to the manufacturer's instructions (Abcam ab112133). Fluorescence readings were obtained using a BD LSR II flow cytometer. The experiment included negative untreated controls and cells treated with 10 µM of carbonyl cyanide *m*-chlorophenyl hydrazone (CCCP) for 15 min as positive controls.

Cell imaging studies

PC3 prostate adenocarcinoma cells were seeded in 8-well microscopy chambers (20000 cells/well; 200 µl phenol red free DMEM medium), left to attach for 48 h, and then treated for another 4 h with IC_{50} concentrations of **Ir-TEMPO1** (16 µM) and **Ir-TEMPO2** (0.5 µM), respectively. Either lysosomes or mitochondria were labelled with LysoTracker® Green DND-26 (50 nM; 30 min at 37; λ_{ex}= 504 nm/λ_{em}= 511 nm) or MitoTracker® Green FM (400 nM; 30 min at 37°C; λex= 490 nm/λem= 516 nm). Cells were then washed with phosphate buffered saline (PBS) and fresh phenol red free medium to remove excess complex not taken up by cells. Cells were then imaged using a Zeiss LSM710 confocal microscope. Images were processed using FIJI ImageJ package.[7]

Synthesis

Synthesis of Ligand **L1**:

The synthesis of **L1** was carried out by reacting 4'-methyl-2,2'-bipyridine-4-carboxylic acid with 4-aminoTEMPO (4-amino-2,2,6,6-tetramethylpiperidine-1-oxyl) using EDC (N-(3-dimethylaminopropyl)-N′-ethylcarbodiimidehydrochloride) coupling. 4'-Methyl-2,2'-bipyridine-4-carboxylic acid (50 mg, 0.233mmol) was treated with EDC (43.45 mg, 0.280 mmol), N-hydroxysuccinimide (32.24mg, 0.280 mmol) and N,N-diisopropylethylamine(50 µL, 0.233 mmol) in 10 mL dimethylformamide (DMF) solvent. The reaction mixture was allowed to stir under nitrogen atmosphere overnight, then 4-aminoTEMPO (39.95 mg, 0.233 mmol) was separately dissolved in 5 mL of DMF and this solution was added to the reaction mixture slowly under constant stirring and the reaction was continued for 24 h at room temperature. The progress of the reaction was monitored from time to time by TLC (thin layer chromatography). The pure compound was isolated by silica column chromatography using methanol and dichloromethane as eluent in a 2:98 v/v solvent mixture. Yield: 52.4%. Anal. Calculated for **L1** [$C_{21}H_{27}N_4O_2$.0.5 H_2O]: C, 67.00; H, 7.50; N, 14.88; Found C, 66.77; H, 7.19; N, 14.75. HRMS: $[M+Na]^+$ Calculated: 368.2207; Found: 368.2208.

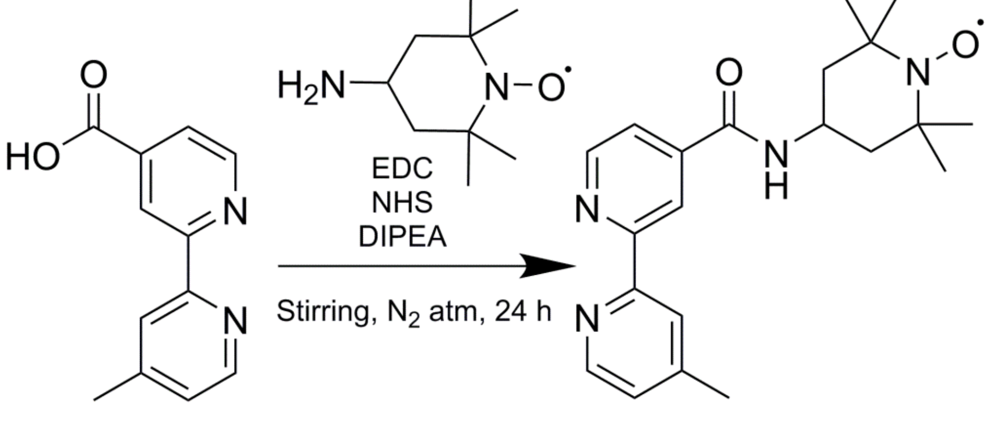

Scheme S1: Synthesis of ligand **L1**

Synthesis of Ligand **L2**:

The synthesis of **L2** followed a similar synthetic procedure as for **L1**. 2,2′-Bipyridine-4,4′-dicarboxylic acid (50 mg, 0.204 mmol) was reacted with EDC (79.40 mg, 0.512 mmol), N-hydroxysuccinimide (58.91 mg, 0.512 mmol) and N,N-diisopropylethylamine (72 µL, 0.409 mmol) in 10 mL dimethylformamide (DMF) solvent. Then 4-aminoTEMPO (77.09 mg, 0.450 mmol) was dissolved in 5 mL of DMF and slowly added in to the reaction mixture. The reaction was carried out under a nitrogen atmosphere for 24 h at room temperature. The compound was purified using a silica column eluted with methanol and dichloromethane 3:98 v/v solvent system. Yield: 62.5%. Anal. Calculated for **L2** [$C_{30}H_{42}N_6O_4$. 0.5 H_2O]: C, 64.38; H, 7.74; N, 15.02; Found C, 64.35; H, 7.66; N, 15.09. HRMS: $[M+Na]^+$ Calculated: 573.3159, found: 573.3152.

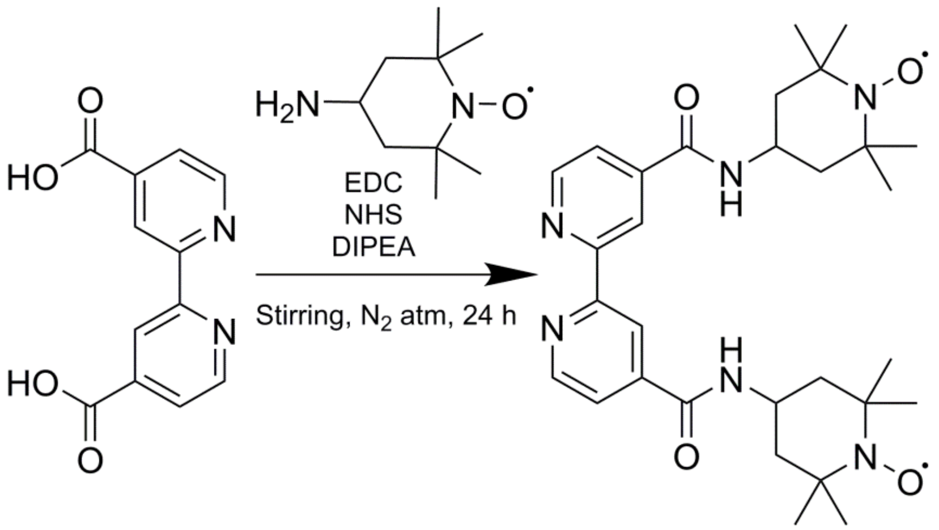

Scheme S2: Synthesis of ligand **L2**

Synthesis of [Ir (N-C)$_2$L1]PF$_6$ (**Ir-TEMPO1**)

Cyclometallated iridium dimer [Ir(N-C)$_2$(μ-Cl)]$_2$ was synthesised by following a reported literature procedure.[8] **Ir-TEMPO1** was synthesised by reacting **L1** (8.335 mg, 0.022 mmol) with [Ir(N-C)$_2$(μ-Cl)]$_2$(23.2 mg, 0.0216 mmol). Prior to this [Ir(N-C)$_2$(μ-Cl)]$_2$was treated with 2 mol equiv AgPF$_6$(10.94 mg, 0.0432 mmol) in 10 mL methanol: dichloromethane (1:1 v/v) solvent mixture. The reaction was stirred at room temperature for 3 h. The silver chloride which precipitated was removed by centrifugation and the yellow coloured supernatant was reacted with **L1** at 65°C for 8 h. The colour of the reaction mixture changed from yellow to red. The volume of the solvent was reduced to 1 mL by using a rotary evaporator, and then diethyl ether was added slowly, giving a pale red-coloured precipitate. Yield: 67%. Anal. Calculated

for **Ir-TEMPO1** [$C_{43}H_{43}N_6F_6Ir_1PO_2.H_2O$]: C, 50.09; H, 4.40; N, 8.15; Found C, 49.74; H, 4.18; N, 8.01. HRMS: $[M]^+$ Calculated: 868.3074, found: 868.3081.

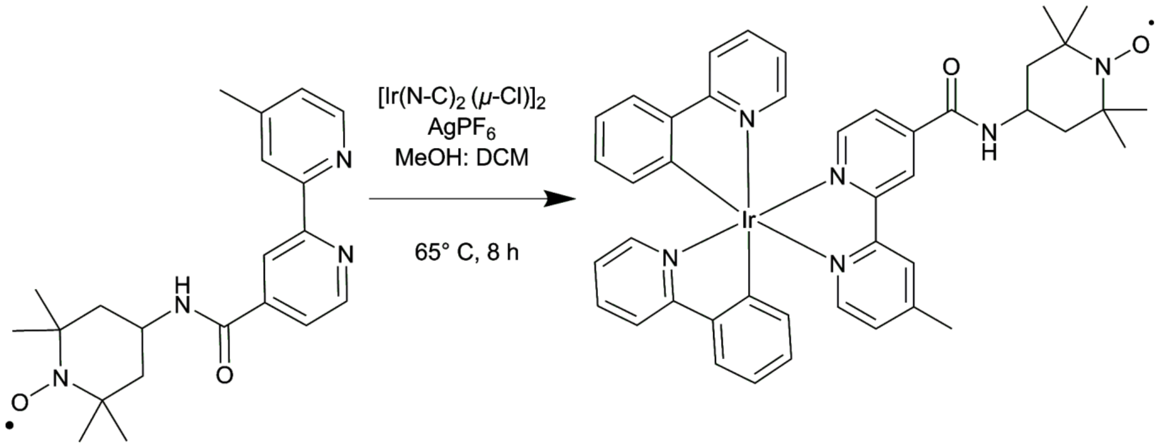

Scheme S3: Synthesis of **Ir-TEMPO1**

Synthesis of [$Ir(N\text{-}C)_2$ L2]PF_6 (**Ir-TEMPO2**):

Ir-TEMPO2 was synthesised by following a similar protocol as for **Ir-TEMPO1,** where **L2** (25 mg, 0.045 mmol) was used instead of **L1**. Yield: 69%. Anal. Calculated for **Ir-TEMPO2** [$C_{52}H_{58}N_8F_6Ir_1PO_4.H_2O$]: C, 51.44; H, 4.98; N, 9.23; Found C, 51.06; H, 4.75; N, 9.07. HRMS: $[M]^+$ Calculated: 1051.4209, found: 1051.4214.

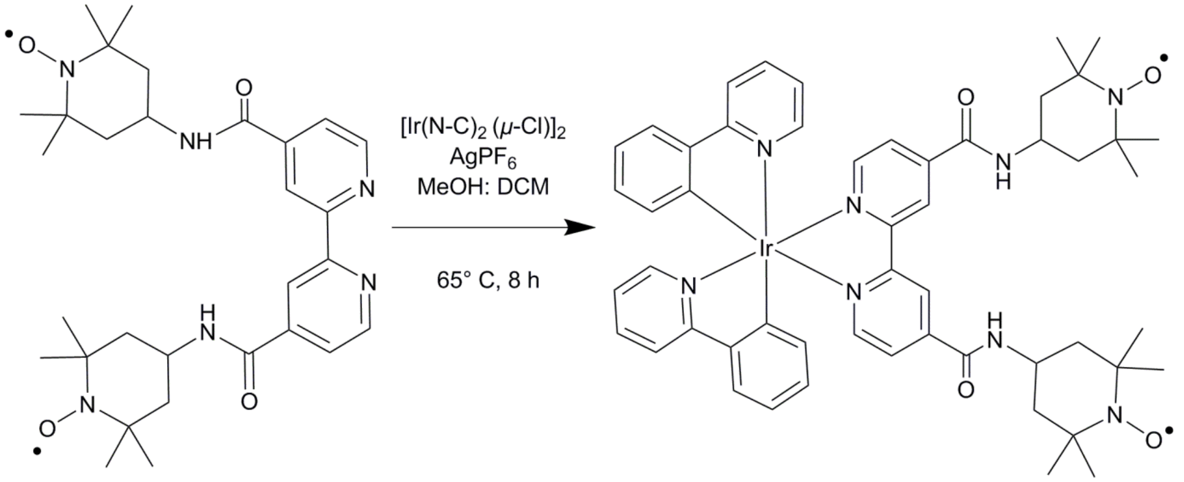

Scheme S4: Synthesis of **Ir-TEMPO2**

Table S1: Global lifetime analysis of **Ir-TEMPO2** in DMSO as a function of excitation and emission wavelength. The global χ^2 was 0.980; the global χ^2 for a monoexponential decay was 6.00.

λ_{exc} (nm)	λ_{em} (nm)	τ_1 (µs)	%	τ_2 (µs)	%
**450**	580	0.165	16.81	0.068	83.19
**450**	640	0.165	5.98	0.068	94.02
**450**	700	0.165	3.28	0.068	96.72
**480**	580	0.165	16.50	0.068	83.50
**480**	640	0.165	5.38	0.068	94.62
**480**	700	0.165	3.08	0.068	96.92
**510**	580	0.165	7.39	0.068	92.61
**510**	640	0.165	2.75	0.068	97.25
**510**	700	0.165	1.30	0.068	98.70

Table S2: Crystal structure refinement data for [$C_{52}H_{58}N_8O_4Ir_1.PF_6$] **(Ir-TEMPO2)**

Identification code	vv1
Empirical formula	$C_{52}H_{58}F_6IrN_8O_4P$
Formula weight	1196.23
Temperature/K	150(2)
Crystal system	orthorhombic
Space group	$Cmc2_1$
a/Å	21.7439(8)
b/Å	20.2524(4)
c/Å	11.8321(3)
α/°	90
β/°	90
γ/°	90
Volume/Å^3	5210.5(3)
Z	4
ρ_{calc}g/cm^3	1.525
μ/mm^{-1}	2.667
F(000)	2416.0
Crystal size/mm^3	0.24 × 0.06 × 0.06 orange block
Radiation	MoK$_\alpha$ (λ = 0.71073)
2Θ range for data collection/°	5.294 to 64.878
Index ranges	$-32 \leq h \leq 30$, $-28 \leq k \leq 29$, $-17 \leq l \leq 17$
Reflections collected	34944
Independent reflections	8874 [R_{int} = 0.0823, R_{sigma} = 0.1022]
Data/restraints/parameters	8874/247/413
Goodness-of-fit on F^2	1.029

Final R indexes [I>=2σ (I)]	R_1 = 0.0626, wR_2 = 0.1242
Final R indexes [all data]	R_1 = 0.1464, wR_2 = 0.1537
Largest diff. peak/hole / e Å^{-3}	0.80/-1.68
Flack parameter	0.001(5)

Table S3: Selected bond lengths for **Ir-TEMPO 2**

Atom	Atom	Length/Å	Atom	Atom	Length/Å
Ir1	N3	2.136(8)	Ir1	C30	1.883(10)
Ir1	N3	2.136(8)	Ir1	N31	2.063(11)
Ir1	N19	2.035(10)	Ir1	C42	2.204(12)

Symmetry: ^{1}1-*x*, +*y*, +*z*

Table S4: Selected bond angles for **Ir-TEMPO 2**

Atom	Atom	Atom	Angle/˚	Atom	Atom	Atom	Angle/˚
N3	Ir1	N31	76.8(5)	C30	Ir1	C42	88.0(7)
N31	Ir1	C42	92.9(6)	C30	Ir1	N31	176.0(6)
N3	Ir1	C42	168.6(6)	C30	Ir1	N3	102.5(6)
N19	Ir1	N3	96.7(6)	C30	Ir1	N19	83.5(7)
N19	Ir1	N31	92.6(5)	C30	Ir1	N31	99.7(8)
N19	Ir1	N31	165.0(7)	C37	C42	Ir1	112.5(9)
N19	Ir1	C42	88.7(7)	C41	C42	Ir1	127.5(9)
N31	Ir1	N31	84.3(6)	C2	N3	Ir1	125.8(7)
N31	Ir1	N3	97.0(5)	C4	N3	Ir1	115.9(7)
N31	Ir1	C42	76.8(6)	C20	N19	Ir1	129.8(9)
C24	N19	Ir1	110.2(9)				

Symmetry: ^{1}1-*x*, +*y*, +*z*

Table S5: Hydrogen bonds for **Ir-TEMPO 2**

D	H	A	d(D-H)/Å	d(H-A)/Å	d(D-A)/Å	D-H-A/°
C5	H5	F16[1]	0.95	2.60	3.097(16)	112.8
N8	H8	F14[2]	0.88	2.12	2.948(13)	156.9
C10	H10B	F14[1]	0.99	2.54	3.220(15)	125.9
C14	H14B	F13	0.99	2.67	3.196(14)	113.8

Symmetry of A: [1]1-*x*,-*y*,-1/2+*z*; [2]1-*x*, +*y*, +*z*

Computational studies:

The LFMM calculations were carried out using DommiMOE[9], an extended version of the Molecular Operating Environment version 2015.1001.[10] The Merck Molecular FF (MMFF94)[11-14] distributed with MOE was augmented with additional parameters to handle Ir-N(pyridyl) and Ir-C(benzyl) interactions. An additional atom-matching rule was defined for the three-coordinate TEMPO N atom in order to assign it the normally two-coordinate N—O (nitroxyl) atom type, together with an out-of-plane term to generate the small amount of pyramidalisation apparent in the experimental X-ray structure. LFMM parameters for metal-ligand bonding were based on the force field developed for Ru-arene systems.[15] Minor adjustments were made to the M-L Morse function parameters to give a reasonable match between calculated and experimental Ir-L distances. Ir^{3+} is a low-spin d^6 centre which has a strong preference for octahedral coordination which is easily generated by the chosen LFMM parameters. Since the conformational flexibility is in the amido-TEMPO substituents coming off the back of the bipyridyl ligand, there seemed little to be gained by further optimising the LFMM parameters. Partial atomic charges were based on the modified MMFF94 charge scheme in MOE which assigns charges to saturated alkanes as compared to the original MMFF94 scheme where C and H charges would be zero. A full list of partial atomic charges for $[Ir\text{-}TEMPO2]^+$ is given below.

Stochastic searches started from the observed X-ray conformation where both TEMPO chains are extended. The $[PF_6]^-$ anions were removed. Electrostatic cut-offs were disabled and solvation was treated via the R-field approach implemented in MOE. The number of steps for the stochastic search was progressively increased from 1000 to 2000 and then 4000.

The 2000 and 4000 step searches also generate a number of other conformations within a few kcal mol^{-1} of the global minimum. Two of these, together with the global LFMM minimum

and the X-ray conformation were then subjected to DFT optimisations. The spin restricted DFT calculations employed the Becke-Perdew (BP) gradient corrected functional or the B3LYP hybrid functional, a Zeroth Order Regular Approximation (ZORA) relativistic correction, def2-SVP or def2-TZVP basis sets, Grimme's D3 dispersion correction and a conductor-like screening model solvation field appropriate for dichloromethane. Geometry optimisations only used the BP functional with B3LYP restricted to single-point energy evaluations.

Table S6: Calculated energies (kcal mol^{-1}) and nitroxyl N···N separations (Å) for **Ir-TEMPO2** complex. Source indicates the starting geometry. Conformer **1** (X-ray start) is the crystallographically-observed geometry while the starting geometries for Conformers **2–4** were generated from LFMM stochastic searches. SC min was common to all three conformational searches while Conformers **3** and **8** from the 4000-step search (SC3) were selected based on their N···N separations.

Conf	*Source*	*Method*	*Erel(kcal)*	*r(N...N)*
1	X-ray start	BP/ZORA/SVP/D3	11.3	9.88
2	LFMM SC min	BP/ZORA/SVP/D3	6.2	5.53
3	LFMM SC3 conf 3	BP/ZORA/SVP/D3	5.8	4.88
4	LFMM SC3 conf 8	BP/ZORA/SVP/D3	0.0	7.00
1	X-ray start	BP/ZORA/SVP/D3/COSMO(CH2Cl2)	3.5	9.27
2	LFMM SC min	BP/ZORA/SVP/D3/COSMO(CH2Cl2)	5.7	5.65
3	LFMM SC3 conf 3	BP/ZORA/SVP/D3/COSMO(CH2Cl2)	7.5	4.99
4	LFMM SC3 conf 8	BP/ZORA/SVP/D3/COSMO(CH2Cl2)	0.0	6.94
1	X-ray start	E: B3LYP/ZORA/TZVP/D3/COSMO(CH2Cl2)	0.0	9.27
2	LFMM SC min	E: B3LYP/ZORA/TZVP/D3/COSMO(CH2Cl2)	9.3	5.65
3	LFMM SC3 conf 3	E: B3LYP/ZORA/TZVP/D3/COSMO(CH2Cl2)	12.4	4.99
4	LFMM SC3 conf 8	E: B3LYP/ZORA/TZVP/D3/COSMO(CH2Cl2)	3.7	6.94
1	X-ray start	E: BP/ZORA/TZVP/D3/COSMO(CH2Cl2)	0.0	9.27
2	LFMM SC min	E: BP/ZORA/TZVP/D3/COSMO(CH2Cl2)	6.0	5.65
3	LFMM SC3 conf 3	E: BP/ZORA/TZVP/D3/COSMO(CH2Cl2)	8.8	4.99
4	LFMM SC3 conf 8	E: BP/ZORA/TZVP/D3/COSMO(CH2Cl2)	1.1	6.94
1	X-ray start	BP/ZORA/TZVP/D3/COSMO(CH2Cl2)	0.0	9.13
2	LFMM SC min	BP/ZORA/TZVP/D3/COSMO(CH2Cl2)	5.9	5.66
3	LFMM SC3 conf 3	BP/ZORA/TZVP/D3/COSMO(CH2Cl2)	8.4	5.30
4	LFMM SC3 conf 8	BP/ZORA/TZVP/D3/COSMO(CH2Cl2)	1.1	6.91

Additional MOE FF MMFF94 parameters

```
type  Ir+3  Ir    'Ir+3 (d6)'

[rules]
N=O   match '[N;X3]=[#8]'

[ang]
*     Ir+3  NPYD  Car   120.040.0     0.0000 0 0
```

```
*     Ir+3  Car   Car   120.571   40.5167  -16.2500 0 0

[tor]
*       Car      Ir+3    Car      Car       0.      0.     0.      0.      0.
*       NPYD     Ir+3    Car      Car       0.      0.     0.      0.      0.

[oop]
Ir+3  NPYD  Car   Car    2.00
O=    N=O   C     C     -1.00
```

LFMM parameters

```
# ad hoc FF for Sadler Ircyclometallated complexes
[Morse]
Ir+3 NPYD 2.30 100 1.30
Ir+3 Car  2.20 120 1.20
%

[ll]
Ir+3 NPYD 100 6
Ir+3 Car 100 6
%

[esig]
Ir+3 NPYD 0 0 -179272 843858 -671272 0 0
Ir+3 Car 0 0 -179272 843858 -671272 0 0
%
[epix]
Ir+3 NPYD 0 0 0 0 0 0 0
Ir+3 Car 0 0 0 0 0 0 0
%

[epiy]
Ir+3 NPYD -7038.09 0 121185 -156909 0 0 0
Ir+3 Car -7038.09 0 121185 -156909 0 0 0
%

[exds]
Ir+3 NPYD 0 0 0 -52433.5 222321 -166430 0
Ir+3 Car 0 0 0 -52433.5 222321 -166430 0
%

[pair]
Ir+3 NPYD 0 0 0 0 0 0 0
Ir+3 Car 0 0 0 0 0 0 0
%
```

Atom types, partial atomic charges and van der Waals parameters

Atom	Q	R	Eps	m	N
Ir+3 (Ir 1)	2.2690	1.7000	0.1060	12	6
Car (C 2)	-0.1500	1.7250	0.0680	12	6
HC (H 3)	0.1500	1.4850	0.0220	12	6
Car (C 4)	-0.1540	1.7250	0.0680	12	6

HC	(H	5)	0.1500	1.4850	0.0220	12	6
NPYD	(N	6)	-0.2010	1.8680	0.0720	12	6
Car	(C	7)	-0.0040	1.7250	0.0680	12	6
Car	(C	8)	-0.1500	1.7250	0.0680	12	6
HC	(H	9)	0.1500	1.4850	0.0220	12	6
Car	(C	10)	0.0860	1.7250	0.0680	12	6
C=	(C	11)	0.5440	1.9920	0.0680	12	6
O=	(O	12)	-0.5700	1.7460	0.0760	12	6
NC=O	(N	13)	-0.7300	1.9450	0.0720	12	6
HN2	(H	14)	0.3700	1.3070	0.0220	12	6
C	(C	15)	0.2200	1.9690	0.0680	12	6
HC	(H	16)	0.0800	1.4850	0.0220	12	6
C	(C	17)	-0.1600	1.9690	0.0680	12	6
HC	(H	18)	0.0800	1.4850	0.0220	12	6
HC	(H	19)	0.0800	1.4850	0.0220	12	6
C	(C	20)	0.1220	1.9690	0.0680	12	6
N=O	(N	21)	0.3200	2.0770	0.0720	12	6
O=	(O	22)	-0.5640	1.7460	0.0760	12	6
C	(C	23)	0.1220	1.9690	0.0680	12	6
C	(C	24)	-0.1600	1.9690	0.0680	12	6
HC	(H	25)	0.0800	1.4850	0.0220	12	6
HC	(H	26)	0.0800	1.4850	0.0220	12	6
C	(C	27)	-0.2400	1.9690	0.0680	12	6
HC	(H	28)	0.0800	1.4850	0.0220	12	6
HC	(H	29)	0.0800	1.4850	0.0220	12	6
HC	(H	30)	0.0800	1.4850	0.0220	12	6
C	(C	31)	-0.2400	1.9690	0.0680	12	6
HC	(H	32)	0.0800	1.4850	0.0220	12	6
HC	(H	33)	0.0800	1.4850	0.0220	12	6
HC	(H	34)	0.0800	1.4850	0.0220	12	6
C	(C	35)	-0.2400	1.9690	0.0680	12	6
HC	(H	36)	0.0800	1.4850	0.0220	12	6
HC	(H	37)	0.0800	1.4850	0.0220	12	6
HC	(H	38)	0.0800	1.4850	0.0220	12	6
C	(C	39)	-0.2400	1.9690	0.0680	12	6
HC	(H	40)	0.0800	1.4850	0.0220	12	6
HC	(H	41)	0.0800	1.4850	0.0220	12	6
HC	(H	42)	0.0800	1.4850	0.0220	12	6
NPYD	(N	43)	-0.2010	1.8680	0.0720	12	6
Car	(C	44)	-0.1540	1.7250	0.0680	12	6
HC	(H	45)	0.1500	1.4850	0.0220	12	6
Car	(C	46)	-0.1500	1.7250	0.0680	12	6
HC	(H	47)	0.1500	1.4850	0.0220	12	6
Car	(C	48)	-0.1500	1.7250	0.0680	12	6
HC	(H	49)	0.1500	1.4850	0.0220	12	6
Car	(C	50)	-0.1500	1.7250	0.0680	12	6
HC	(H	51)	0.1500	1.4850	0.0220	12	6
Car	(C	52)	-0.0040	1.7250	0.0680	12	6
Car	(C	53)	0.0000	1.7250	0.0680	12	6
Car	(C	54)	-0.1500	1.7250	0.0680	12	6
HC	(H	55)	0.1500	1.4850	0.0220	12	6
Car	(C	56)	-0.1500	1.7250	0.0680	12	6
HC	(H	57)	0.1500	1.4850	0.0220	12	6
Car	(C	58)	-0.1500	1.7250	0.0680	12	6
HC	(H	59)	0.1500	1.4850	0.0220	12	6
Car	(C	60)	-0.1500	1.7250	0.0680	12	6
HC	(H	61)	0.1500	1.4850	0.0220	12	6
Car	(C	62)	-0.2140	1.7250	0.0680	12	6
NPYD	(N	63)	-0.2010	1.8680	0.0720	12	6
Car	(C	64)	-0.1540	1.7250	0.0680	12	6
HC	(H	65)	0.1500	1.4850	0.0220	12	6

Car	(C 66)	-0.1500	1.7250	0.0680	12	6
HC	(H 67)	0.1500	1.4850	0.0220	12	6
Car	(C 68)	-0.1500	1.7250	0.0680	12	6
HC	(H 69)	0.1500	1.4850	0.0220	12	6
Car	(C 70)	-0.1500	1.7250	0.0680	12	6
HC	(H 71)	0.1500	1.4850	0.0220	12	6
Car	(C 72)	-0.0040	1.7250	0.0680	12	6
Car	(C 73)	0.0000	1.7250	0.0680	12	6
Car	(C 74)	-0.1500	1.7250	0.0680	12	6
HC	(H 75)	0.1500	1.4850	0.0220	12	6
Car	(C 76)	-0.1500	1.7250	0.0680	12	6
HC	(H 77)	0.1500	1.4850	0.0220	12	6
Car	(C 78)	-0.1500	1.7250	0.0680	12	6
HC	(H 79)	0.1500	1.4850	0.0220	12	6
Car	(C 80)	-0.1500	1.7250	0.0680	12	6
HC	(H 81)	0.1500	1.4850	0.0220	12	6
Car	(C 82)	-0.2140	1.7250	0.0680	12	6
Car	(C 83)	-0.1500	1.7250	0.0680	12	6
HC	(H 84)	0.1500	1.4850	0.0220	12	6
Car	(C 85)	-0.1540	1.7250	0.0680	12	6
HC	(H 86)	0.1500	1.4850	0.0220	12	6
NPYD	(N 87)	-0.2010	1.8680	0.0720	12	6
Car	(C 88)	-0.0040	1.7250	0.0680	12	6
Car	(C 89)	-0.1500	1.7250	0.0680	12	6
HC	(H 90)	0.1500	1.4850	0.0220	12	6
Car	(C 91)	0.0860	1.7250	0.0680	12	6
C=	(C 92)	0.5440	1.9920	0.0680	12	6
O=	(O 93)	-0.5700	1.7460	0.0760	12	6
NC=O	(N 94)	-0.7300	1.9450	0.0720	12	6
HN2	(H 95)	0.3700	1.3070	0.0220	12	6
C	(C 96)	0.2200	1.9690	0.0680	12	6
HC	(H 97)	0.0800	1.4850	0.0220	12	6
C	(C 98)	-0.1600	1.9690	0.0680	12	6
HC	(H 99)	0.0800	1.4850	0.0220	12	6
HC	(H 100)	0.0800	1.4850	0.0220	12	6
C	(C 101)	0.1220	1.9690	0.0680	12	6
N=O	(N 102)	0.3200	2.0770	0.0720	12	6
O=	(O 103)	-0.5640	1.7460	0.0760	12	6
C	(C 104)	0.1220	1.9690	0.0680	12	6
C	(C 105)	-0.1600	1.9690	0.0680	12	6
HC	(H 106)	0.0800	1.4850	0.0220	12	6
HC	(H 107)	0.0800	1.4850	0.0220	12	6
C	(C 108)	-0.2400	1.9690	0.0680	12	6
HC	(H 109)	0.0800	1.4850	0.0220	12	6
HC	(H 110)	0.0800	1.4850	0.0220	12	6
HC	(H 111)	0.0800	1.4850	0.0220	12	6
C	(C 112)	-0.2400	1.9690	0.0680	12	6
HC	(H 113)	0.0800	1.4850	0.0220	12	6
HC	(H 114)	0.0800	1.4850	0.0220	12	6
HC	(H 115)	0.0800	1.4850	0.0220	12	6
C	(C 116)	-0.2400	1.9690	0.0680	12	6
HC	(H 117)	0.0800	1.4850	0.0220	12	6
HC	(H 118)	0.0800	1.4850	0.0220	12	6
HC	(H 119)	0.0800	1.4850	0.0220	12	6
C	(C 120)	-0.2400	1.9690	0.0680	12	6
HC	(H 121)	0.0800	1.4850	0.0220	12	6
HC	(H 122)	0.0800	1.4850	0.0220	12	6
HC	(H 123)	0.0800	1.4850	0.0220	12	6

BP/ZORA/SVP/D3/COSMO optimised Cartesian coordinates

Conf1

```
123
Coordinates from ORCA-job Ir_conf1_sol
Ir  -11.01782992422305      6.63973082752359      3.15140448939441
  C   -7.50568453942364      4.01562473909046      2.54928402212797
  H   -6.41742201542067      4.13200532704946      2.51945485918177
  C   -8.33100950553596      5.10673853623133      2.82536899854116
  H   -7.92164568119151      6.10045739452734      3.03874463357257
  N   -9.68013083555777      5.00465384519120      2.84476387941337
  C   -10.26749992985530      3.79967109259754      2.57910089637169
  C   -9.48954373716222      2.66045297162270      2.31569414568666
  H   -9.98192648448982      1.71150228137900      2.08185181685732
  C   -8.08931378311374      2.75735474581760      2.30576912594593
  C   -7.18703614922922      1.59323183751782      1.96904920508610
  O   -6.07490773882256      1.78585781138949      1.46340279201419
  N   -7.69750602364402      0.36073084209569      2.24457713684294
  H   -8.55060308323935      0.29021286119298      2.79900567124343
  C   -6.97478894602702     -0.87479068298823      1.96277667736600
  H   -6.19021452693674     -0.59433797085045      1.23740416489422
  C   -7.92214650679911     -1.91797944036701      1.36642548072504
  H   -8.42458665712026     -1.51430939996487      0.46867250373999
  H   -8.71207762062867     -2.14053196234581      2.11172267747529
  C   -7.21275696564318     -3.23005815646358      0.97924871271146
  N   -6.30067665684963     -3.67941270688511      2.08978474795373
  O   -5.83780172516629     -4.86955063305074      1.99042115274287
  C   -5.51847556310199     -2.73425085182431      2.96307209234616
  C   -6.33444238933032     -1.45454475584083      3.22959595877717
  H   -7.13999556317694     -1.67873456635952      3.95648536814803
  H   -5.66914837342115     -0.70755220760479      3.69900455212500
  C   -4.17373430085418     -2.41414595540997      2.27169281608605
  H   -4.30130734327771     -1.79182812517342      1.37034793999257
  H   -3.68249142412675     -3.35720452782628      1.97761029743438
  H   -3.51196771319561     -1.87165593737382      2.96917072634222
  C   -5.25584690216688     -3.45979251711825      4.29369824837160
  H   -4.67045549231043     -4.37658516714037      4.12213253629160
  H   -6.20850662457620     -3.73736469403135      4.77850434429967
  H   -4.69511501979179     -2.79506073734826      4.97321828294778
  C   -8.26186869557028     -4.33694115418005      0.77827201239003
  H   -8.96309425775149     -4.04208864357267     -0.02133508537053
  H   -8.83501088004349     -4.50095416266553      1.70791841444495
  H   -7.77294058555539     -5.28284479175706      0.49779829511269
  C   -6.38001813673675     -3.06104786068263     -0.31150394900662
  H   -5.69359073796833     -2.19971738542264     -0.25748372639194
  H   -7.05056215111992     -2.91001140022418     -1.17543968145078
  H   -5.78324685561940     -3.97327446280831     -0.48112909849198
  N   -10.72757834149761      7.16264662755458      1.19018207838542
  C   -11.39929086906992      6.61387340240924      0.15050334485737
  H   -12.14176743277524      5.85017826400708      0.40006852218538
  C   -11.17108227907178      7.00247510759494     -1.17039369603025
  H   -11.74127115050680      6.53284495755917     -1.97828304732100
  C   -10.20741086789875      7.99636674220380     -1.42370261789544
  H   -10.00267107234443      8.32682623593856     -2.44843549050792
  C   -9.51274604044837      8.56055412987325     -0.34934455789699
  H   -8.75961022802772      9.33592293069289     -0.52180094187024
  C   -9.78007513289234      8.13786838039724      0.96837135558082
  C   -9.14183394109028      8.64277078792767      2.18364082130571
  C   -8.12791020299841      9.62680688111317      2.19123987170641
  H   -7.77051387116580     10.06651517550895      1.25166472997083
```

C	-7.57133046401736	10.04707362330567	3.40629141448849
H	-6.78438021202990	10.81051927584839	3.41571749593012
C	-8.03080378672255	9.48345488369786	4.61459118206059
H	-7.59917657592370	9.81435912156126	5.56825082662031
C	-9.03972671827135	8.50424065344762	4.61037899885196
H	-9.38096858514797	8.08782243019143	5.56588382855493
C	-9.61333798063740	8.05824891376112	3.40098728649791
N	-11.30834824802070	6.34505876331549	5.15958628760514
C	-10.62834970155505	5.43829203579357	5.90023953387811
H	-9.87857313605435	4.84298767219480	5.37077188583128
C	-10.85661523491643	5.27331018150717	7.26722679160847
H	-10.27920885373639	4.52994854114115	7.82615329578371
C	-11.82921410793650	6.07764168473865	7.88997864311460
H	-12.03406604743061	5.97642207187929	8.96189964055048
C	-12.53223759777409	7.01222541633441	7.12348438215439
H	-13.29202675793688	7.64988047781353	7.58638519313665
C	-12.26451672789926	7.14498146814653	5.74603632123570
C	-12.91043587445307	8.08140116631908	4.82675334959347
C	-13.93344446032258	8.97933807320124	5.20583895050253
H	-14.29312991993385	9.00973362042043	6.24198617506744
C	-14.49608730840277	9.83950653049340	4.25387027752376
H	-15.29010707524123	10.53750107113453	4.54465280773121
C	-14.03340748377645	9.80248918971727	2.92217027203365
H	-14.46969046104015	10.47873616505935	2.17545621706847
C	-13.01524630641362	8.91055020891931	2.54188608969143
H	-12.67165313985121	8.90794431062541	1.50037891888298
C	-12.43551738517182	8.02884281212305	3.47852819513230
C	-14.50799775726620	3.97832037895865	2.57191075044341
H	-15.59782695171367	4.08149720711455	2.55274224869422
C	-13.69169048707733	5.09030427786789	2.78472854099766
H	-14.10960385120805	6.08917540250515	2.95293287940074
N	-12.34126359462967	5.00513520695445	2.78491985681574
C	-11.74352137535699	3.79343802629036	2.58068213818204
C	-12.51179384911882	2.63567451338690	2.37721868729335
H	-12.01105191718603	1.68144336168199	2.18785924958334
C	-13.91289349838424	2.71671039182858	2.37853689815782
C	-14.80484167508383	1.52691887395832	2.11095044607296
O	-15.92454479657317	1.68168876427293	1.60918551794405
N	-14.27649645978141	0.31627860757856	2.44457099845196
H	-13.41164189352212	0.28470610058511	2.98427283882112
C	-14.98188892460242	-0.94264816539694	2.23246807864301
H	-15.82818455332923	-0.69241722412830	1.56789376453968
C	-14.05525641028255	-1.96845398846981	1.57457897722295
H	-13.63554415313582	-1.56320085703133	0.63596946454371
H	-13.20383088493984	-2.15945572349737	2.25824410154546
C	-14.75633482780615	-3.30379563508679	1.25866660972487
N	-15.56482106097965	-3.76301964255713	2.44277538218355
O	-16.00173929629363	-4.96600923796643	2.39307857655987
C	-16.30028620559440	-2.82824659061043	3.36637760042550
C	-15.50253641294558	-1.52285725253279	3.55247496685113
H	-14.63587591136564	-1.71551959525915	4.21547365005446
H	-16.14916939808668	-0.78863196345235	4.06608231431690
C	-17.70479660845789	-2.55398400347115	2.78247335755061
H	-17.66783732665887	-1.93972781712759	1.86736449049890
H	-18.19087741065433	-3.51391148826375	2.53872801973203
H	-18.32361408757024	-2.02130499620784	3.52545092213661
C	-16.43368229458395	-3.54332557161699	4.72164325228303
H	-17.00388518171785	-4.47851665218031	4.60793098268619

```
  H   -15.43722595749325     -3.78745512911731      5.13045466873801
  H   -16.95694701582041     -2.88599624011027      5.43728327213512
  C   -13.69655755688444     -4.38497909343155      0.98656192716719
  H   -13.06923408669541     -4.08207525043437      0.13057134629129
  H   -13.04712834919216     -4.52180204636727      1.86927321400392
  H   -14.18011111588514     -5.34702122490966      0.75605948536556
  C   -15.69310489357768     -3.17275501862080      0.03672407758799
  H   -16.39478738023752     -2.32789493364214      0.13579049600003
  H   -15.09756981317894     -3.01651939514343     -0.87966804214229
  H   -16.27801514894936     -4.10148061776785     -0.07448877539014
```

Conf 2

```
123
Coordinates from ORCA-job Ir_conf2_sol
Ir  1.87854216501910     -1.24612831944682      3.78589621051981
  C   2.06400185682200     -2.09623224653977     -0.46120648287900
  H   2.62872721820584     -2.74500256407416     -1.13884436654477
  C   2.32233397812711     -2.11284491961912      0.90945890353303
  H   3.08240208903835     -2.77047655828350      1.33978031872296
  N   1.64711832942053     -1.31787577594046      1.77918470275637
  C   0.68124443084977     -0.47686219981528      1.28614228532843
  C   0.36826187586768     -0.43402786575841     -0.07777393998932
  H   -0.44178775525880      0.22805285390116     -0.40407507118005
  C   1.06320054398785     -1.24793318953354     -0.98121448460555
  C   0.79953353217770     -1.26104329361956     -2.47060446520344
  O   1.46439883723396     -1.99138341284893     -3.21959683777005
  N   -0.19117546656485     -0.43548323253140     -2.90011951812169
  H   -0.72389002972551      0.15271182531854     -2.24969991589065
  C   -0.58369682834331     -0.30987954545882     -4.29876129533407
  H   -0.12772435489029     -1.16477940076193     -4.82987007047485
  C   -0.06660071814801      1.01393442100011     -4.87748356076985
  H   -0.45082634248816      1.83764467279661     -4.24530142917032
  H   1.03588109760543      1.05128543517420     -4.81372720087491
  C   -0.48565312134439      1.26909867698625     -6.33588838587526
  N   -1.95318065877964      0.98353995659270     -6.50823498560104
  O   -2.48641592677500      1.40385146171744     -7.59447169417294
  C   -2.66322894067541     -0.15042482341716     -5.81784934135319
  C   -2.11235893674601     -0.34228743319419     -4.39395468388663
  H   -2.51015876352755      0.45286307518010     -3.73392311411496
  H   -2.49675396119850     -1.29837042771486     -3.99472346087654
  C   -4.15048408223136      0.23359180519920     -5.73410201116424
  H   -4.27415492064642      1.17782273378096     -5.17470615314047
  H   -4.70874538788610     -0.56089887702778     -5.20964853279178
  H   -4.57385532384362      0.36737564643059     -6.74168840577556
  C   -2.50072427000616     -1.43293256329640     -6.66424736656255
  H   -2.76641689672771     -1.21642865936688     -7.71296031836642
  H   -3.17348545964997     -2.22108827432004     -6.28348607638399
  H   -1.46996040585155     -1.82333991228038     -6.63580578605963
  C   0.31889627280916      0.39130257047616     -7.32083233951380
  H   1.37210907833307      0.72143070261193     -7.34284109740813
  H   -0.10604928813041      0.49570231733763     -8.33370454007283
  H   0.30329366363875     -0.67539803647197     -7.04262053490196
  C   -0.27141301326073      2.75476032864261     -6.67216449656713
  H   0.79135172659319      3.01591398890133     -6.53011981092349
  H   -0.88191623446843      3.39398735969790     -6.00999458448912
  H   -0.55768786778326      2.96052067242514     -7.71526236910644
  N   2.12797357359358     -0.98922149729943      5.82066623401600
  C   1.46349996759332     -1.71333619628033      6.75467542908814
```

H	0.75251102176333	-2.45092297007860	6.37034841517717
C	1.66575057062044	-1.52809900383531	8.12324920759777
H	1.10184420388957	-2.13558265382070	8.83835997280678
C	2.59553120753721	-0.56030925576948	8.54329281937848
H	2.78098005557090	-0.38839914800939	9.60978195470178
C	3.28382538492143	0.18286780502729	7.57978157343804
H	4.01318655487030	0.94052178066048	7.88274243555504
C	3.04638075487840	-0.03590493699510	6.20765074147795
C	3.69838000869180	0.68138398526865	5.10686829199047
C	4.64938858187143	1.71009486351250	5.29884951618325
H	4.94557327358800	2.02200605726799	6.30826411470106
C	5.22187350263031	2.34423179483186	4.18746490488120
H	5.96017094979459	3.14208240656712	4.33190168382350
C	4.84437790502424	1.95294770205918	2.88660368966913
H	5.29122776464516	2.45183183303928	2.01650238741364
C	3.89903359076527	0.92714894510826	2.69903205835151
H	3.62498433267391	0.63863218095778	1.67477914530215
C	3.31011700731405	0.26967500797419	3.79794934415104
N	3.26355396697598	-2.77581558206181	3.88709046787632
C	4.60391302119170	-2.57839784930541	3.83576774390449
H	4.92973675886870	-1.54070992500294	3.71652860957870
C	5.51576990267273	-3.63157050122361	3.92238870310345
H	6.58880594153694	-3.42070457063079	3.87208030037211
C	5.02339671469003	-4.94010561118342	4.07346067949294
H	5.70997251750548	-5.79141533396496	4.14569436598698
C	3.64138227382669	-5.14279803456568	4.13069447307903
H	3.23475496764825	-6.15198864075936	4.24873262018526
C	2.75643125477523	-4.04966753809300	4.03688971909538
C	1.29279079409682	-4.13496674360502	4.07635608946831
C	0.58325408456345	-5.35193596962015	4.19910372248807
H	1.11490054733429	-6.30900556090189	4.27178247498895
C	-0.81823939754122	-5.34027702833834	4.22550445425126
H	-1.37227933845818	-6.28180705501642	4.32054485015929
C	-1.51033678711965	-4.11583745994840	4.12807258231240
H	-2.60814960085749	-4.10753388891464	4.14537024123257
C	-0.80086328348882	-2.90615622824885	4.00861231966725
H	-1.36452836248437	-1.96547164994043	3.93857528961842
C	0.60823540270035	-2.88759225423859	3.98323050119958
C	-1.18692205054450	1.77686406854822	4.32983737522117
H	-1.65797212477336	2.30435002066253	5.16587637064980
C	-0.18069988819165	0.84142475164095	4.57905940364893
H	0.16449930009019	0.63409210640018	5.59556150730197
N	0.41878338569766	0.13434893942309	3.58355050990676
C	0.01984147793547	0.34856798150858	2.28879593588749
C	-0.95621347702086	1.29978371669669	1.98120123210225
H	-1.26317224441392	1.42600225129662	0.93954235905060
C	-1.57297737208282	2.03873618411329	2.99895819480087
C	-2.70490381571783	2.96946874917474	2.63153216642973
O	-3.72381922227720	3.06975227883612	3.32275578769111
N	-2.51456907791690	3.57889990264212	1.42583215842893
H	-1.57048188806443	3.55860989516615	1.04638138381808
C	-3.55234693806917	4.18537850800261	0.58717653737840
H	-3.98393525324393	5.06087826476476	1.10830530563256
C	-4.68682680706386	3.19777525165763	0.27208233002846
H	-5.17301554147407	2.87877290705534	1.20907506772615
H	-5.44492806369243	3.75289848574839	-0.30823377392909
C	-4.21807131237277	1.94090030870396	-0.51755415933517
N	-2.82356033980864	2.15791382651459	-1.04684578750335

```
  O   -2.15961146613197     1.10231035717898    -1.36423289178002
  C   -2.32003157376224     3.47381579190210    -1.57339137290149
  C   -2.92072756505957     4.64361199308671    -0.73040606665650
  H   -3.70891777251445     5.15520089744908    -1.30860852301580
  H   -2.13327248787098     5.39564875133665    -0.54391552266653
  C   -2.71508540146388     3.61763226879159    -3.06052625391030
  H   -2.36970928256103     2.74442603535689    -3.63975594493716
  H   -3.80473377838219     3.71558009939200    -3.18802036424720
  H   -2.24172124667892     4.52196577988238    -3.48070457572143
  C   -0.78147508084625     3.44967045545889    -1.47687482811615
  H   -0.35744666704315     2.64393994038265    -2.09540958377557
  H   -0.38326628960943     4.41379635822250    -1.83331883095438
  H   -0.41830260771634     3.30012099567910    -0.44403889611013
  C   -5.15649411082271     1.64560912386732    -1.70932236302375
  H   -4.76561955609144     0.79820722230863    -2.29890630147234
  H   -5.27220590161430     2.51960093747242    -2.37046098303809
  H   -6.15665054116694     1.37501810118933    -1.32870864702720
  C   -4.18163684033042     0.70376123106283     0.39814018956779
  H   -3.53228302031404     0.86139779510872     1.27344272146145
  H   -5.20230868599661     0.51109866186822     0.76845404420670
  H   -3.82327220247070    -0.18099144302519    -0.14952273152630
```

Conf 3

```
123
Coordinates from ORCA-job Ir_conf3_sol
Ir  1.37096572858444    -3.23393354948318    -2.50642030817662
  C   4.31069484207178    -0.55296939876782    -0.80620535598059
  H   5.39428345371996    -0.40779809493996    -0.86658640187045
  C   3.71356077379981    -1.61893010742249    -1.47815777564582
  H   4.30710744477515    -2.34180082531282    -2.04426107020246
  N   2.36607852356190    -1.80098691802082    -1.49591887166289
  C   1.56651354197918    -0.92883387587582    -0.79990353074246
  C   2.12139408189119     0.10830052648941    -0.04274933790050
  H   1.45150648341932     0.78365744075613     0.49724752288373
  C   3.50590585280192     0.32829125839814    -0.05515698148008
  C   4.05431228686302     1.59349173425436     0.55864894212578
  O   4.94491803983678     2.24546116697374     0.00229619421399
  N   3.39514964367390     1.98366066705521     1.68402833471261
  H   2.79024181407426     1.29730217092563     2.12985116913135
  C   3.37759490200950     3.34732773606442     2.21991856097932
  H   4.41266872687032     3.65750842545646     2.45512250605166
  C   2.52937020483530     3.38610804426792     3.49140138238211
  H   2.56172300237956     4.42148950706461     3.87209795198056
  H   2.96957039960074     2.74852137055066     4.27832248151297
  C   1.04957366904437     2.95178749490826     3.26011726153397
  N   0.72081200215559     3.03467169293787     1.79235802272317
  O   -0.31014805097364     2.37271953286327     1.39561717203346
  C   1.29469568068763     4.06811341679729     0.85440556217965
  C   2.78845801621264     4.34201399619669     1.20779862782913
  H   3.39065200209855     4.32976034584542     0.28368302236506
  H   2.89616645097927     5.35128689493668     1.64271308485404
  C   0.45992829578456     5.36287565096390     0.96622958143411
  H   -0.60153018387289     5.15914377207407     0.74684453910537
  H   0.53767487345735     5.81696025136952     1.96737964071778
  H   0.83069724267466     6.09850212720788     0.23145521989488
  C   1.17240216838537     3.52435897923744    -0.57984865375559
  H   1.77876979080923     2.61599056461278    -0.72509545399331
  H   1.54899070628930     4.29079050218345    -1.27751121772614
```

H	0.12602589879982	3.29688273660700	-0.83559489264318
C	0.09110285867407	3.85821682303755	4.06312939555175
H	0.23844123495518	4.92250749729688	3.81849056594850
H	0.29454477573617	3.73152786397235	5.14090824176205
H	-0.96332064378853	3.59211870264756	3.88413446720864
C	0.82213378960382	1.49259014722081	3.70797100035002
H	-0.23567870188969	1.21144924122486	3.61134549578286
H	1.12010832280138	1.39537562772916	4.76491426419643
H	1.40956468913673	0.75889603282779	3.12707601282396
N	0.25112297895421	-4.56190683550152	-3.62611600957886
C	-0.18830230306887	-5.75501572201141	-3.15552065393782
H	0.06830380119580	-5.98277354593267	-2.11658252681411
C	-0.92897346803831	-6.63866915848937	-3.94209756639556
H	-1.26457231539104	-7.58899175046367	-3.51470795020474
C	-1.22239786187233	-6.27780876743226	-5.26920143290245
H	-1.80088349840697	-6.94727748567152	-5.91612893803248
C	-0.76537279054670	-5.04940062439915	-5.75549028575496
H	-0.98067643125214	-4.74732118119703	-6.78512318531758
C	-0.02235221470256	-4.18633575068673	-4.92480909156224
C	0.50196757764911	-2.87342134442105	-5.31487401030990
C	0.30434438113037	-2.30098737284486	-6.59269855990595
H	-0.26620677604626	-2.83153486732438	-7.36527782335474
C	0.84038522873610	-1.03807132270632	-6.87953183216188
H	0.68929773872845	-0.59056483970471	-7.86917450656994
C	1.57173108099142	-0.34694824413767	-5.89188439801582
H	1.98813134369796	0.64403145146406	-6.11582020329015
C	1.76880944224890	-0.92078977164695	-4.62208635430664
H	2.34543415166416	-0.36232960574869	-3.87160272179648
C	1.24409364944273	-2.19031178739785	-4.30698252691805
N	3.04375083874797	-4.31335012810677	-3.06427614897272
C	3.70940295542144	-4.11624132382830	-4.22890321781535
H	3.32135492998542	-3.32043672352740	-4.87194948129102
C	4.82539606605974	-4.87441853558070	-4.58656171854361
H	5.33085484272123	-4.67436055708693	-5.53677112346773
C	5.26763969231883	-5.88004954839688	-3.70865793880629
H	6.13963399219425	-6.49583166161450	-3.95723829815716
C	4.57814160918876	-6.08609259423620	-2.51012802790457
H	4.90260740460887	-6.86379719912530	-1.81181973612452
C	3.45669865575516	-5.29499538024274	-2.18829168272664
C	2.65678273554383	-5.41213259566188	-0.96431104601499
C	2.93510156846655	-6.33935789892843	0.06623399875231
H	3.78521253652987	-7.02881101259400	-0.01064699606134
C	2.11817392698209	-6.37961620710584	1.20480105174508
H	2.33007195059154	-7.09775436825316	2.00596562370371
C	1.02664436975271	-5.49423256520001	1.31521125077904
H	0.38881628523091	-5.52454009055561	2.20844387299595
C	0.75095823490609	-4.57382232771946	0.28649375436365
H	-0.10795520338202	-3.89726541405861	0.39538734329961
C	1.55252406840974	-4.51483009884470	-0.87135576761983
C	-2.51538990026687	-1.57444359162294	-1.60770237767434
H	-3.56181403815212	-1.76579038842626	-1.86677064525268
C	-1.50472132738463	-2.40469203472840	-2.08433060145520
H	-1.72279462304128	-3.26426427747915	-2.72344220036528
N	-0.19699687208206	-2.18089575501723	-1.79596987723098
C	0.13189728405923	-1.11939251154263	-0.98840530279568
C	-0.84789422453672	-0.25913217323325	-0.46912871083614
H	-0.53334744479792	0.57164696091761	0.17477727344758
C	-2.19528666582303	-0.46447674137835	-0.79646977566998

```
  C   -3.33117251305808      0.42921943207673     -0.35623286005986
  O   -4.50539983273870      0.11163271219841     -0.60261075166851
  N   -2.99824629674820      1.57601729803143      0.29034770868655
  H   -2.02173293153294      1.82573281810287      0.47100284752042
  C   -4.02967007259745      2.50625636864755      0.76428926225569
  H   -4.74586296373112      2.63186793914614     -0.06858971385716
  C   -4.83502919330623      1.93686146072755      1.95504948030283
  H   -4.94732786414974      0.84695018510833      1.82772825911053
  H   -5.85905370479343      2.34537253648553      1.90847011357666
  C   -4.24773752459296      2.22029231581565      3.34643665075169
  N   -3.92891259579172      3.67930499204368      3.47260145500364
  O   -3.54727697633585      4.10434162645627      4.61898955516392
  C   -3.95259099059069      4.61183679926765      2.29789942218104
  C   -3.35156194849694      3.85366528978852      1.09564030538069
  H   -3.35877254836477      4.51403433290290      0.20987958835083
  H   -2.28917808729816      3.66226460910367      1.33871372082337
  C   -3.06116569414121      5.81771749801931      2.62634540488657
  H   -2.03183211486338      5.49798544442949      2.85355835297688
  H   -3.03652635288044      6.49620401747934      1.75637953898073
  H   -3.44815414590503      6.36732777789433      3.49820627652867
  C   -5.39338856681208      5.11070279205300      2.04198364314239
  H   -6.08317810777643      4.30245522827790      1.75320748096193
  H   -5.38942466971056      5.85972606193653      1.23000669447663
  H   -5.78804721741600      5.59002635260704      2.95476156195334
  C   -2.94820138869001      1.42569009076912      3.58859245745575
  H   -2.18403993823239      1.65554416291769      2.82807310439488
  H   -2.54300299042722      1.68483032641550      4.58159706178857
  H   -3.15326389507204      0.34132253711021      3.55946044966719
  C   -5.28669107419515      1.87170172491717      4.42854365290565
  H   -5.51198788230221      0.79143486517650      4.40405175277191
  H   -6.22507124019309      2.43023828861648      4.26273078858742
  H   -4.89247067488642      2.13584021177694      5.42331744533980
```

Conf 4

```
123
Coordinates from ORCA-job Ir_conf4_sol
Ir  4.01887026019987     -0.82007323235297     -0.48850476456956
  C   1.63398042112316      2.14193887207742      1.61612653158193
  H   1.80162047442208      3.08062570587012      2.15447426468617
  C   2.70436821604642      1.45909466370418      1.03524867546195
  H   3.72958526818890      1.83706431753895      1.09106084522578
  N   2.53963095250556      0.28075447136814      0.38158018199593
  C   1.27714480181947     -0.24484692730183      0.28242090245469
  C   0.16567654669169      0.40873127883569      0.82916888858699
  H   -0.82467773592520     -0.04103411311991      0.70231343965708
  C   0.32903474751315      1.61972743890585      1.51403297422034
  C   -0.80683332432265      2.36688170954772      2.18230010007409
  O   -0.55786259552288      3.34206516355562      2.90721985696964
  N   -2.05661299640313      1.88749773544161      1.94256617075290
  H   -2.21091620302405      1.11355761422173      1.28498338763434
  C   -3.28184428095716      2.45851940575050      2.50746761530480
  H   -4.09284968733089      1.82660539008241      2.09913545147461
  C   -3.51358575012891      3.91072531806592      2.07456454026203
  H   -3.46123102722955      3.99484364232862      0.97371438549293
  H   -2.69765055479933      4.52695811375728      2.49173144819335
  C   -4.87519155482066      4.46058372371138      2.53988946252681
  N   -5.10844935423914      4.12662257166586      3.98947077808620
  O   -6.09195396244596      4.72266505365619      4.55400541653807
```

C	-4.65018609893184	2.84808866610755	4.63913793992767
C	-3.30841696993488	2.38684671596604	4.03822772870218
H	-2.48867051817062	3.01842911884948	4.42361002061708
H	-3.11293042591586	1.35293727986778	4.37775972488399
C	-4.45928859921587	3.14377055622897	6.13664049798837
H	-3.70168242568137	3.93424085097103	6.27934410360789
H	-5.40692516819202	3.47960475024252	6.58615644884066
H	-4.11668499313969	2.23095343224056	6.65392765553845
C	-5.75467377482567	1.78207730468893	4.45853982271207
H	-6.72107755925757	2.19772202999720	4.79059822753777
H	-5.52284332272288	0.89024887848546	5.06648989335681
H	-5.86101843668692	1.46154572878472	3.40866511187127
C	-6.04054945576774	3.88149175226800	1.70535735566734
H	-5.98971548976969	4.26346233877621	0.67083089531553
H	-6.99968279857603	4.19311026194690	2.15271161458687
H	-6.02193399747922	2.77920595610781	1.66251374997760
C	-4.86988249932532	5.99439316646490	2.42417504792048
H	-4.06799559188909	6.42497202853560	3.04932434341255
H	-5.83481421963246	6.40887698408469	2.75578758384510
H	-4.69223322833705	6.28573974636413	1.37459691300374
N	3.83561940668334	0.42386856454522	-2.23006194186991
C	2.92002668843421	0.26189553169954	-3.20979838467806
H	2.25685953048064	-0.60572176349176	-3.11793619909685
C	2.82045486673091	1.14091727959980	-4.29134368379529
H	2.06514412766101	0.97137334920238	-5.06556352035689
C	3.70928055153277	2.23043390896035	-4.34920133015579
H	3.66392581491800	2.94227941915290	-5.18155098690269
C	4.65369479329197	2.39944344078153	-3.33099966803975
H	5.34938237962050	3.24398604894929	-3.35861457716790
C	4.70891501826079	1.48110208864668	-2.26127546223549
C	5.64027947540347	1.54003157056746	-1.12721410200441
C	6.62784159829936	2.54341532036789	-0.99553422251343
H	6.73344234665610	3.32279744358877	-1.76030549177519
C	7.48265332240830	2.55006275244566	0.11407779679731
H	8.24773352693565	3.32922595608505	0.21368868786503
C	7.35184464637565	1.54911304549077	1.09746825365711
H	8.02060664170483	1.54866183865695	1.96809687231011
C	6.37193000130046	0.54886246577177	0.97158800323378
H	6.28608486911014	-0.22038632799873	1.74819865476263
C	5.49248529802261	0.52033616352096	-0.13226763817359
N	5.42266086350740	-2.08487900648136	-1.27474355316494
C	5.86362933634662	-2.02543984578596	-2.55401882778489
H	5.42012631111050	-1.24889991398697	-3.18368111485259
C	6.82994227164611	-2.90339580131989	-3.04766562557654
H	7.15396696677445	-2.81870785152682	-4.08986311095409
C	7.36537722354219	-3.87469413509487	-2.18178748196012
H	8.12727946657067	-4.57868472851030	-2.53547574402131
C	6.91512521083653	-3.92926291291081	-0.85901922481998
H	7.31943685350999	-4.67340485015798	-0.16549471941570
C	5.93652286875398	-3.02270402596597	-0.40500181513695
C	5.37469482079654	-2.96221889228537	0.94410085761829
C	5.74823907578249	-3.82979473902350	1.99490912365137
H	6.50064096462998	-4.61170888637818	1.83237831246412
C	5.15553272420825	-3.69328663049083	3.25724265051345
H	5.44458811388351	-4.36352929368083	4.07544342041983
C	4.18696916670161	-2.69006164133760	3.46783769058415
H	3.72081699903305	-2.58213818157935	4.45592611151961
C	3.81422794828633	-1.82503543070708	2.42401491837262

H	3.06292012870283	-1.04994991496703	2.62056537629115
C	4.39988029649518	-1.93619675717329	1.14543388960027
C	1.13301154803175	-3.97470616748949	-1.71897179938980
H	1.15422146123033	-4.92803613131194	-2.25752318780792
C	2.31180831736518	-3.24028244542379	-1.54134852715429
H	3.27215897613383	-3.60637232942544	-1.92022480965547
N	2.33723187487555	-2.04759836120049	-0.90024248008547
C	1.17463015299213	-1.53713479442265	-0.40252027738903
C	-0.03270349670440	-2.22900899303345	-0.53422070723214
H	-0.95594809172172	-1.77915090901135	-0.16213858958968
C	-0.06840947668400	-3.47137120980276	-1.18314033738012
C	-1.41476330600924	-4.13328022245219	-1.37675958273104
O	-1.72442817230454	-4.69605757075086	-2.43139618658729
N	-2.25168247810801	-3.93002992259882	-0.31804159665873
H	-1.81981760744145	-3.62850276085383	0.55295403258245
C	-3.71432699209153	-4.02559503423045	-0.32799294706444
H	-4.01863823600908	-5.08102336621639	-0.46315590975385
C	-4.25392320146020	-3.51631349443217	1.01277809309944
H	-3.87188987883471	-4.13262052734038	1.84629053062714
H	-5.34784463289114	-3.65956049128968	0.99707426166662
C	-3.92157953977909	-2.01747977696179	1.29484635237371
N	-3.49584207640534	-1.33943950955848	0.02189764316541
O	-2.90342453043835	-0.20190777730289	0.13297723127687
C	-4.05743721466264	-1.66491834237303	-1.33925094390180
C	-4.33776154541348	-3.19118180418714	-1.45640024041709
H	-5.42524644743683	-3.38094868488046	-1.42982100730445
H	-3.97450483616785	-3.55381730487880	-2.43220623323410
C	-3.02140443568288	-1.21768358321801	-2.38698345015510
H	-2.07694555672383	-1.77648662218059	-2.29464874839947
H	-2.80345925884310	-0.14327665545726	-2.29089527774909
H	-3.43022306512283	-1.41483043129085	-3.39198497908698
C	-5.35210034590908	-0.84495131599584	-1.53825922264691
H	-5.71705761992896	-0.98117413305932	-2.57103925075963
H	-5.14835343192156	0.22720910490811	-1.37392182742591
H	-6.15168148188696	-1.16768046436519	-0.85148590851650
C	-2.76401442886753	-1.87985162584872	2.30557851360733
H	-2.56165277640336	-0.82262383508710	2.53865875236530
H	-1.82141179617747	-2.32833323292886	1.94412894939154
H	-3.03933277751579	-2.39567708679339	3.24002485880065
C	-5.15227867741153	-1.28197295588306	1.86883533457018
H	-6.03746264520819	-1.39350719736935	1.22273836986617
H	-4.92791307440935	-0.20764854816606	1.98080247902899
H	-5.39942880091065	-1.69210360730324	2.86307120052147

BP/ZORA/TZVP/D3/COSMO optimised Cartesian coordinates

Conf 5

123

Coordinates from ORCA-job Ir_conf5_sol

Ir	4.02299809699163	-0.74818053411060	-0.51392224233421
C	1.63466392292367	2.33496577913954	1.35769284080777
H	1.80262893550497	3.32082839461031	1.78782777362934
C	2.69474414833379	1.62306093297644	0.81672908517100
H	3.70783231656830	2.01883719805207	0.80599558428134
N	2.53892751531849	0.38945682753425	0.28158362986307
C	1.28630078564847	-0.16211385689597	0.27021170235326
C	0.18847863174153	0.51485008478615	0.79707220474800
H	-0.78584021325380	0.02794443701566	0.76167634943316
C	0.34699431397273	1.78343036942872	1.35343545965160

C	-0.78139009405724	2.57732846751534	1.96536368702451
O	-0.54716594303451	3.64168988879631	2.55705334257239
N	-2.02248931056585	2.06186279214658	1.81382290655061
H	-2.16807588320905	1.15676717202284	1.36321557659318
C	-3.21437326575337	2.66129275196790	2.40579475264747
H	-2.99844928824790	3.73065633166647	2.53479057748140
C	-3.51382574026272	2.01005802855760	3.75649262714941
H	-2.66459305612120	2.14675358108207	4.44090040166299
H	-3.62807096956888	0.92588888292193	3.59380489434897
C	-4.77919432308543	2.54821385614437	4.43681756198590
N	-5.90882027692365	2.59524813654201	3.44536868054763
O	-7.08802458952180	2.75651801053212	3.93269581161302
C	-5.72706982251086	2.98795351105057	2.00483400258954
C	-4.39683849680985	2.44177071587314	1.46875411812382
H	-4.49460728312104	1.35873875097770	1.28878390934589
H	-4.20155794946453	2.90883005552940	0.49263055678561
C	-6.87513414784840	2.35639277237870	1.20908499157920
H	-6.89252084213194	1.26723211317203	1.35426742728293
H	-7.84223909417771	2.76685663922256	1.52002829747985
H	-6.72752541564209	2.56479270705712	0.14062281889315
C	-5.79542086893938	4.52183143135490	1.88293083234297
H	-6.69623551148223	4.89461494481106	2.38745824246256
H	-5.84687493922595	4.80039631342018	0.82125548620595
H	-4.91826596178948	5.01390215604447	2.32085622007905
C	-4.54942025461595	3.95473987896848	5.02030092987033
H	-3.84841012659538	3.88873374172314	5.86378588305353
H	-5.50014516196446	4.36465466450439	5.38522793615160
H	-4.12799943865882	4.64877787139532	4.28290394216545
C	-5.18979918291389	1.58632293541784	5.55698563819775
H	-5.43666887775123	0.59708360655706	5.14692744915529
H	-6.06079569991236	1.96695507001735	6.10208393617474
H	-4.35188725738491	1.47535232297469	6.25860541116122
N	3.85880662791204	0.38559376219329	-2.32603748640987
C	2.95460445198128	0.16979593608155	-3.30148066148503
H	2.28324248130431	-0.67608560272286	-3.15610750449176
C	2.87917133144086	0.96781178608457	-4.43485190657795
H	2.13321424485853	0.75577155721103	-5.19898277166685
C	3.77662873562299	2.03385748835369	-4.55845139658486
H	3.74827946474723	2.68216172066575	-5.43435328565859
C	4.70770742434263	2.26111050982101	-3.55137047842428
H	5.41055486987739	3.08859950085307	-3.63116282131320
C	4.74220859858127	1.42512595403869	-2.42569878714681
C	5.66061096780497	1.55161342234350	-1.29417021110919
C	6.64828708883215	2.55026436585566	-1.22319673286297
H	6.76480267685991	3.27112882906146	-2.03401658857727
C	7.48848577649681	2.62703987154886	-0.11566140984700
H	8.25378435414980	3.40203025685254	-0.06294978453021
C	7.34330877267664	1.70223944925551	0.92659450462291
H	8.00084572367556	1.75727328531728	1.79653304690269
C	6.36337498865249	0.70729443776652	0.86113849605700
H	6.26758707668615	-0.00143159595318	1.68468147213062
C	5.49762465628319	0.60734496706398	-0.23970872063239
N	5.43111813637496	-2.05447386844528	-1.20999186968577
C	5.88406461964381	-2.07445416814377	-2.48264197906912
H	5.45172698887976	-1.33881224839289	-3.15679129770701
C	6.84394733111815	-2.97873358698091	-2.91193158716196
H	7.17547615217359	-2.95602392745434	-3.94854881615609
C	7.36465078234366	-3.89508234174873	-1.99254825462891

H	8.12192256404373	-4.61737969717838	-2.29697817936983
C	6.90588284782669	-3.87161826255277	-0.68103901825601
H	7.29881469200731	-4.57270552615013	0.05337654719748
C	5.93363551605651	-2.94249528079224	-0.28917384578065
C	5.36475791333476	-2.80315696190163	1.04476036382435
C	5.72922525686720	-3.60708899511830	2.13806194423920
H	6.47513625747460	-4.39544349036547	2.02312435018521
C	5.13630739287497	-3.39842353063840	3.38097506100144
H	5.41809596111087	-4.01883190009486	4.23222349703635
C	4.17803452288555	-2.38637280966802	3.53042433074895
H	3.71267418946668	-2.22074173239859	4.50418161055666
C	3.81479475608052	-1.58462620984847	2.44405567499218
H	3.07165773306516	-0.79971138174468	2.59595714727346
C	4.39873394574375	-1.76920733183236	1.18171541683567
C	1.16821861028069	-3.98367679911554	-1.51063379923458
H	1.19591625015411	-4.95699607452908	-1.99825296299842
C	2.32607092063006	-3.22114774811070	-1.40904728161590
H	3.27796759319953	-3.58148627334570	-1.79640601788017
N	2.34471755725684	-2.00095816061812	-0.83092461221875
C	1.18841716731548	-1.49506000762913	-0.32230837446197
C	0.00234667343950	-2.22088156105768	-0.37365309191200
H	-0.91408498271109	-1.78040313472937	0.01357152009847
C	-0.01972890066310	-3.48487422365989	-0.96361225432557
C	-1.33389326476470	-4.21435906955012	-1.07326212345600
O	-1.65581259461986	-4.83416065324007	-2.09325607243765
N	-2.12650596826306	-4.03499798608535	0.01189463981477
H	-1.68345962489560	-3.63730301891933	0.83337656746213
C	-3.57521828101608	-4.25041082932712	0.08866334371563
H	-3.79091002562465	-5.32084267885585	-0.04520872788169
C	-4.05935336499864	-3.80442264282812	1.46636636281877
H	-3.56622438619969	-4.38715052713136	2.25696546357058
H	-5.13047960385557	-4.03807118216862	1.52311064783078
C	-3.83786263588986	-2.28775767157143	1.74384898886757
N	-3.52285022247203	-1.57965733505953	0.45691826614326
O	-2.94666834167115	-0.42816756386699	0.54221700646035
C	-4.15037455255822	-1.92180374600719	-0.86676914644211
C	-4.33774738433601	-3.45780086268182	-0.97712892647153
H	-5.39946898654631	-3.71728208288914	-0.86139282125209
H	-4.03635264123467	-3.78355076009475	-1.98024386009323
C	-3.21956750628606	-1.40969465513329	-1.97321455572515
H	-2.23475088787785	-1.89001103280681	-1.93386567031591
H	-3.08306801814679	-0.32531172645565	-1.90382614489092
H	-3.67276169406625	-1.65265185190212	-2.94279301728181
C	-5.49960494819391	-1.18841224555777	-0.98125072309065
H	-5.91127977126503	-1.34957215524715	-1.98704020158940
H	-5.35821553591827	-0.10954760782343	-0.83122404541805
H	-6.23086196515224	-1.56022275063103	-0.25385883196482
C	-2.65506156387816	-2.06522678748691	2.69854279741555
H	-2.53957803532357	-1.00431490334145	2.94580761968145
H	-1.69715632083656	-2.41333843478157	2.28522553532821
H	-2.84093165624383	-2.62327105233404	3.62451242398455
C	-5.09538701231386	-1.66651052977215	2.37909528311053
H	-5.99185051931765	-1.85813405027338	1.77849361065884
H	-4.97094111285152	-0.58157621287462	2.49665720946518
H	-5.25237072290036	-2.10681685063044	3.37335171217894

Table S7: IC_{50} values for $[Ir(N\text{-}C)_2(\mu\text{-}Cl)]_2$, and $[Ir(ppy)_2(bpy)]^+$ (precursor analogues without TEMPO units, and mono- and bis-TEMPO ligands **L1** and **L2**, against A2780 human ovarian, A2780Cis cisplatin-resistant human ovarian, A549 human lung, PC3 human prostate cancer cell lines, and normal MRC5 human lung fibroblast cell line.

Cell lines	IC_{50} (µM)			
	**$[Ir(N\text{-}C)_2(\mu\text{-}Cl)]_2$**	**$[Ir(ppy)_2(bpy)]^+$**	**L1**	**L2**
A2780	>100 µM	>100 µM	N/A	N/A
A2780Cis	>100 µM	>100 µM	N/A	N/A
A549	>100 µM	>100 µM	84 ± 2	75 ± 5
PC3	>100 µM	>100 µM	74 ± 3	84 ± 4

Table S8: Flow cytometry analysis of mitochondrial membrane potential (MMP) in PC3 prostate cancer cells exposed to iridium complexes **Ir-TEMPO1** and **Ir-TEMPO2** at equipotent IC_{50} concentrations by flow cytometry using the JC-10 assay (Abcam).

	**Q1**	**Q2**	**Q3**	**Q4**
Negative control	0.51 ± 0.08	71 ± 4	23 ± 4	5.9 ± 0.6
Positive control*	0.39 ± 0.07	8.2 ± 0.5	85.7 ± 0.9	5.7 ± 0.4
Ir-TEMPO1	0.05 ± 0.02	8 ± 2	85 ± 2	6.7 ± 0.8
Ir-TEMPO2	0.01 ± 0.02	2.4 ± 0.1	88 ± 1	9 ± 1

* Positive control samples, treated with carbonyl cyanide *m*-chlorophenyl hydrazine (CCCP, 10 µM) for 15 min.

^{1}H NMR of L1, L2, Ir-TEMPO1, Ir-TEMPO2:

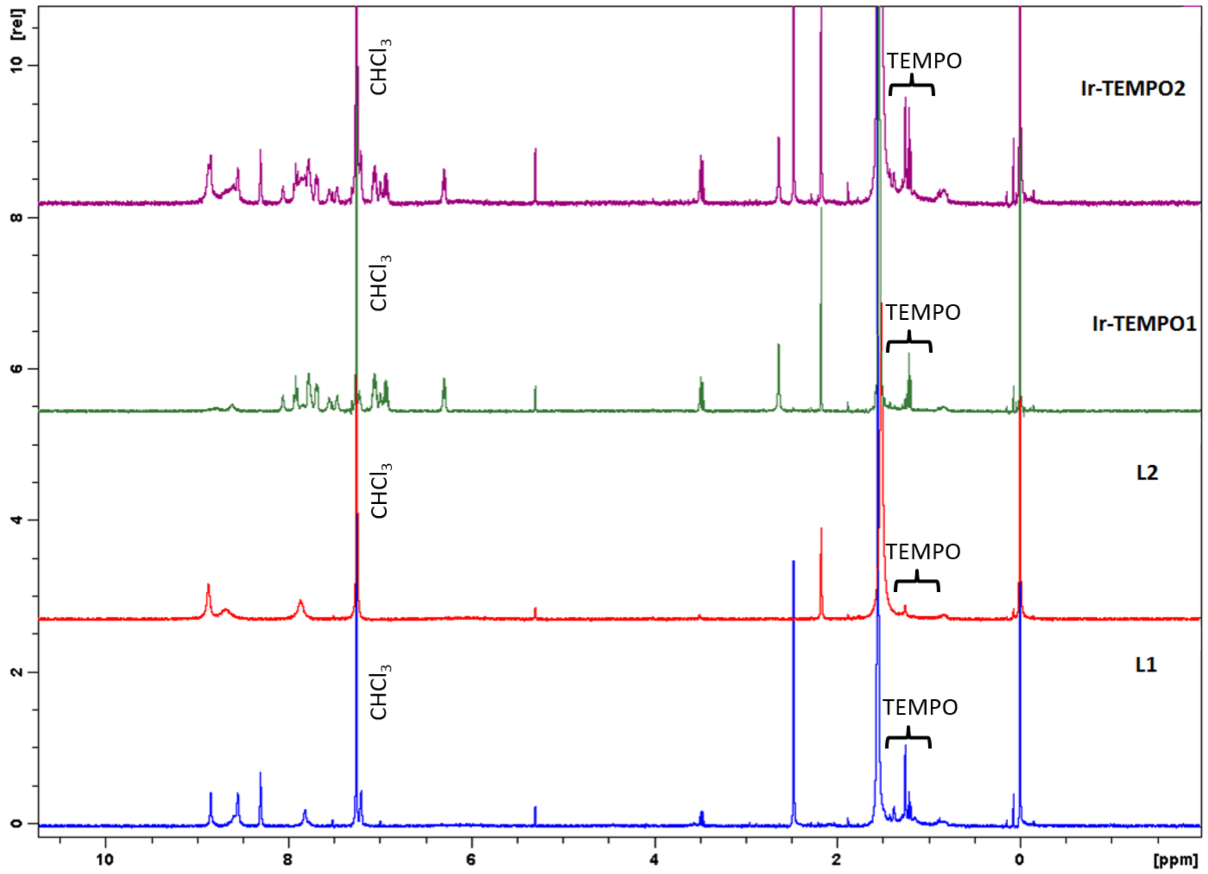

Figure S1: ^{1}H NMR spectra of **L1**, **L2**, **Ir-TEMPO1** and **Ir-TEMPO2** in $CDCl_3$. Line broadening is clearly seen for **L2** and **Ir-TEMPO2.**

HRMS of L1, L2, Ir-TEMPO1 and Ir-TEMPO2:

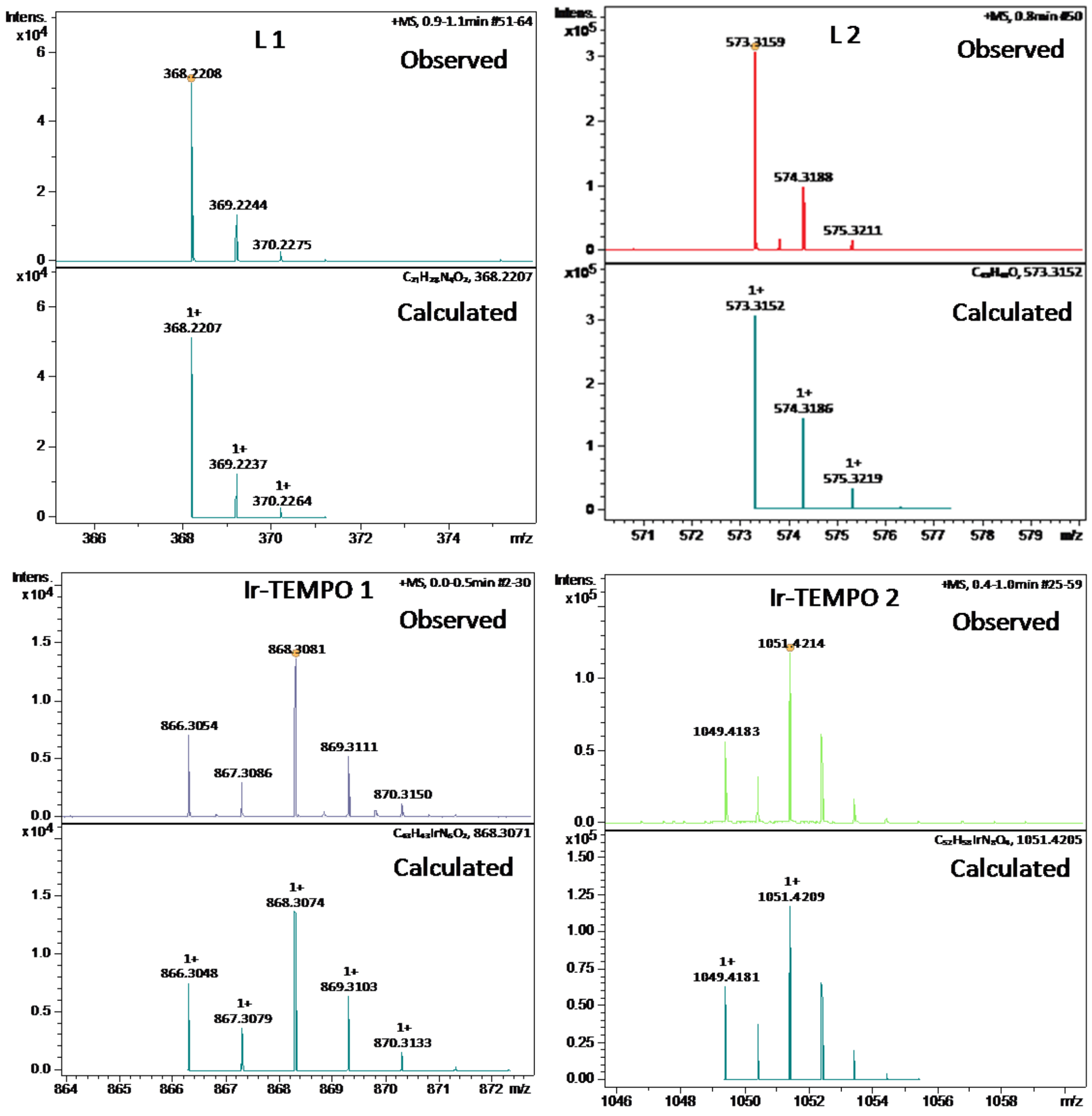

Figure S2: HR-MS spectra of **L1**, **L2**, **Ir-TEMPO1** and **Ir-TEMPO2**. The observed isotopic distribution patterns match well with the calculated isotopic distributions**.**

Additional EPR of L1, L2, Ir-TEMPO1 and Ir-TEMPO2:

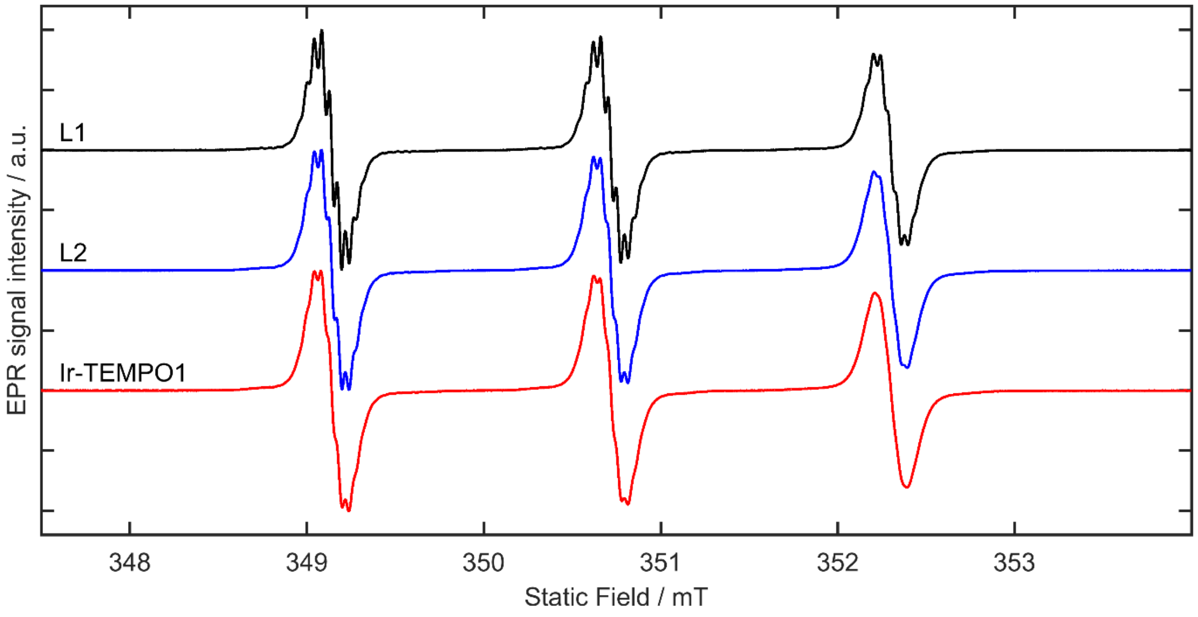

Figure S3: X-band EPR spectra of isolated ligands **L1** and **L2**, and **Ir-TEMPO1** for comparison. All samples were prepared at 0.1 mM concentration in deoxygenated DCM. No indication of a significant electron-electron exchange interaction is seen in any of these systems. Slight differences in the resolution of small proton hyperfine couplings are thought to arise from variation in the efficiency of sample deoxygenation.

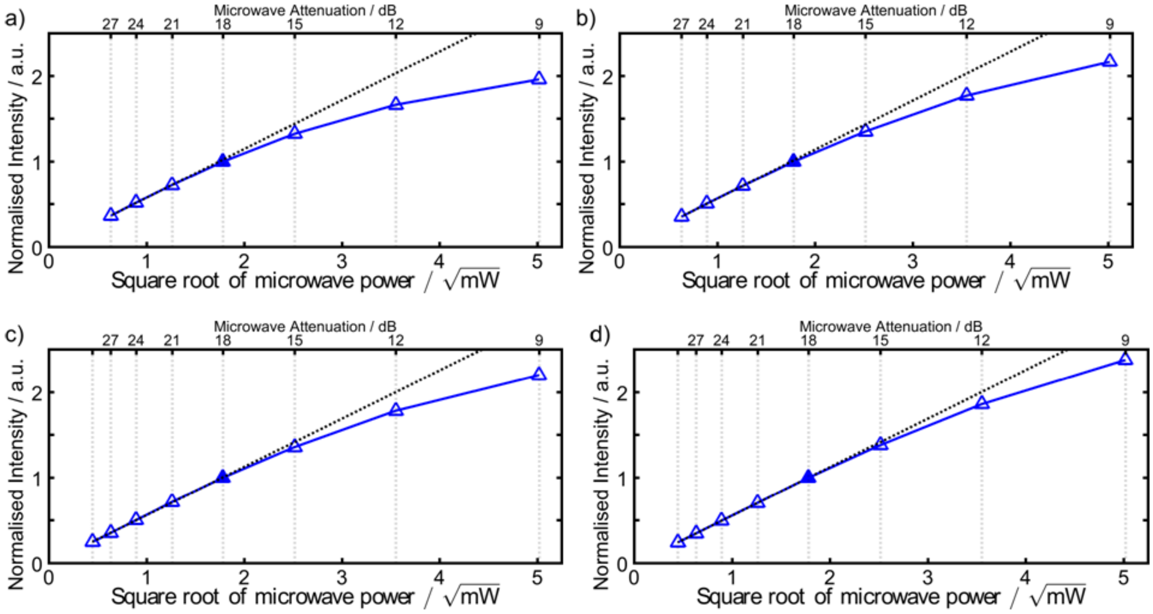

Figure S4: EPR power saturation data for a) **L1**, b) **L2**, c) **Ir-TEMPO1** and d) **Ir-TEMPO2**. Peak-to-peak signal intensity is plotted against the square root of microwave power as calculated from the experimental microwave attenuation setting. Solid lines are a guide to the eye, and the dotted line is a linear fit to the lowest power intensities. The filled symbols indicate the data points for 18 dB microwave attenuation as used for collection of all other EPR spectra presented, and it is clear that at this power the system is operating in the linear regime. Within experimental error, the saturation behaviour is identical for each system, indicating that there are no significant changes in relaxation properties as the structure of the ligand is varied or upon binding to the diamagnetic metal centre.

Simulation method for Ir-TEMPO2:

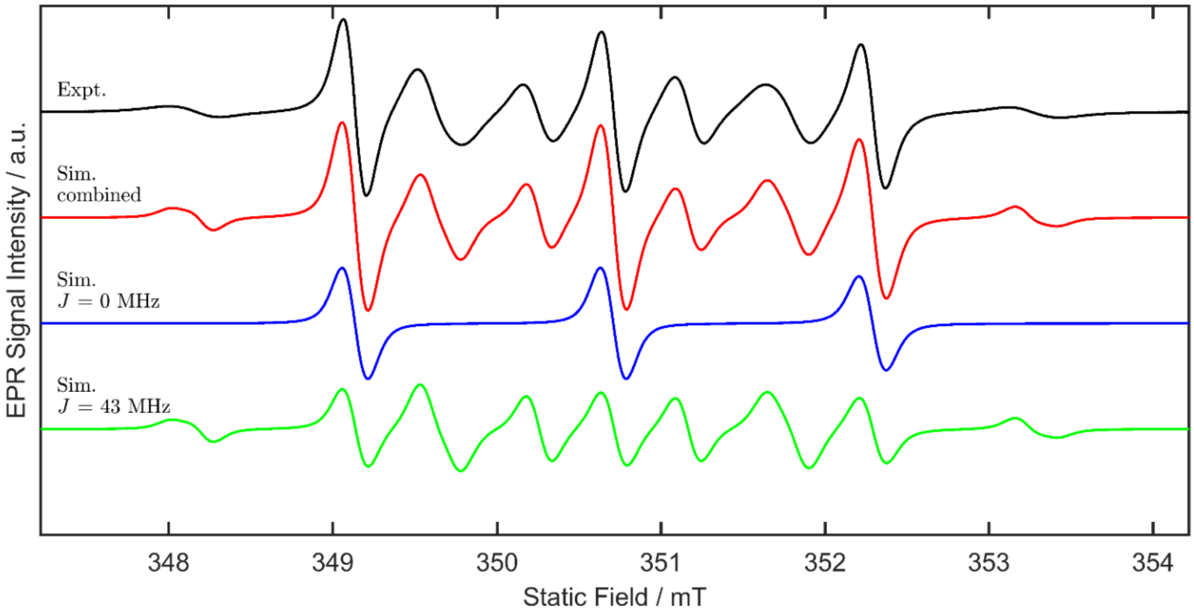

Figure S5: X-band EPR data for **Ir-TEMPO2** along with spectral simulations. The experimental spectrum was approximated as a weighted sum of two individual components having zero and non-zero electron-electron interaction strength *J* as shown.

Confocal Images of **Ir-TEMPO1** and **Ir-TEMPO2** in PC3 prostate cancer cells:

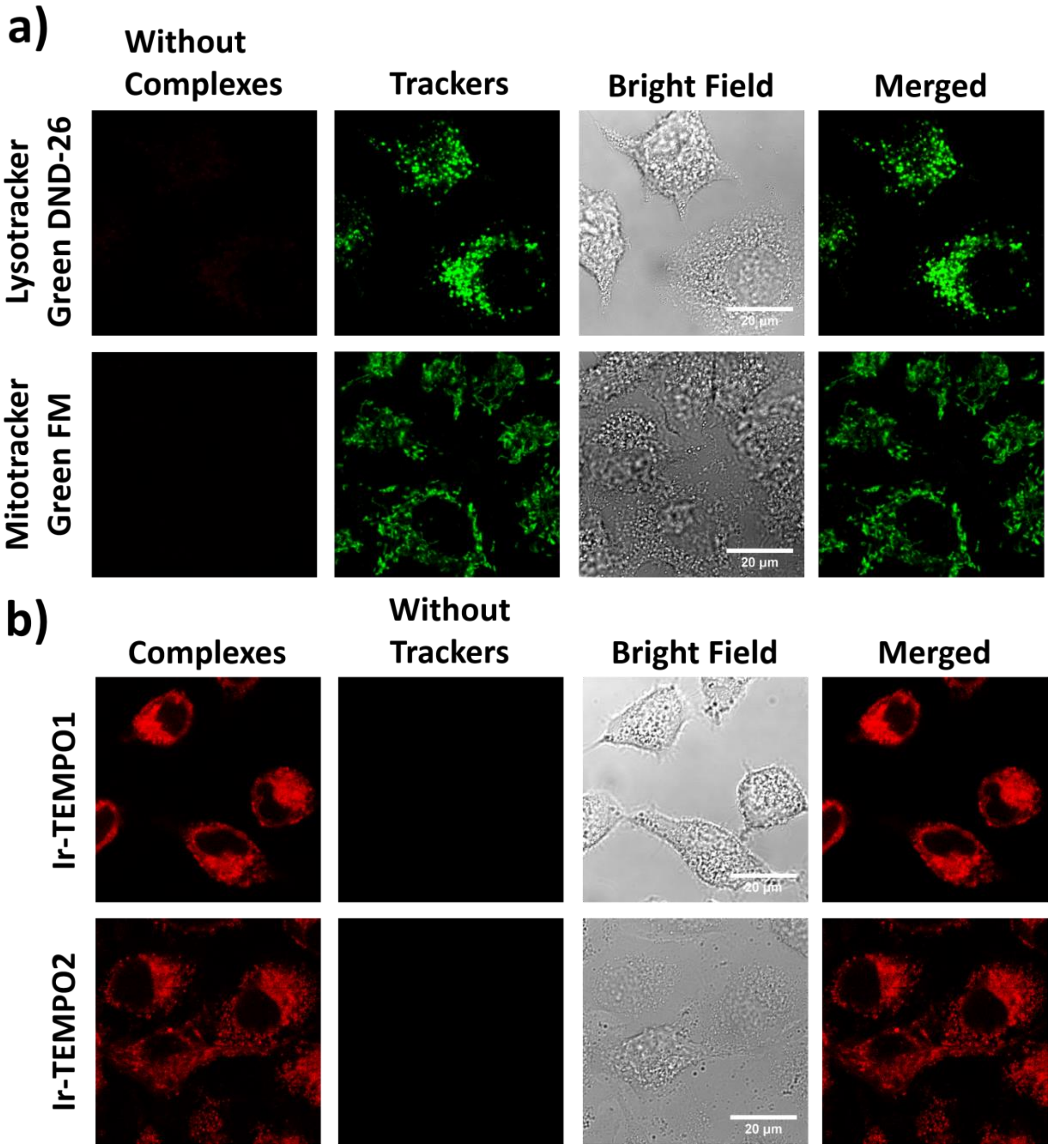

Figure S6: Confocal microscope images of PC3 prostate cancer cells. a) Absence of **Ir-TEMPO1** and **Ir-TEMPO2**. b) Absence of MitoTracker Green FM and LysoTracker Green DND-26.

Mitochondrial membrane potential assay:

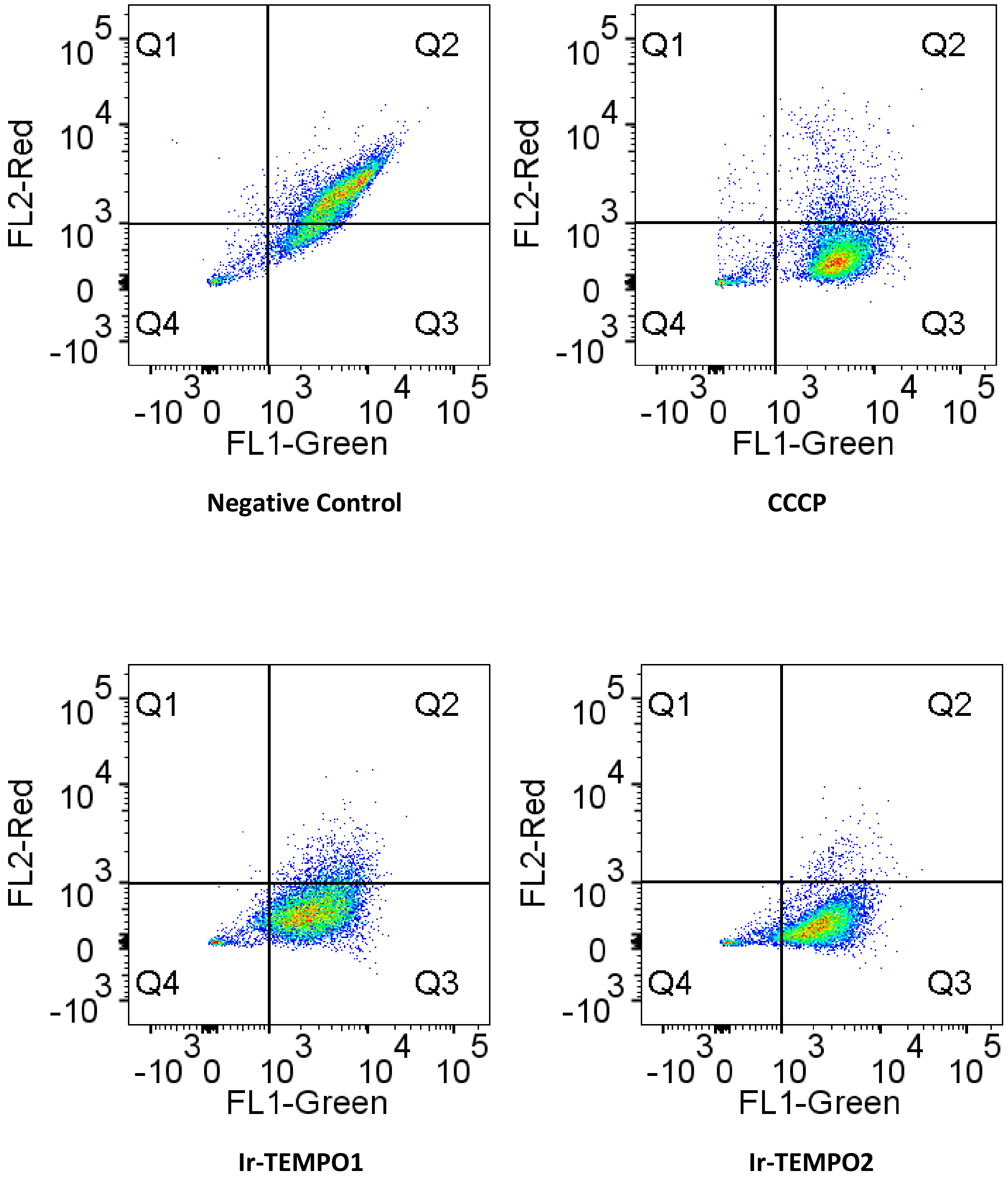

Figure S7: Flow cytometry analysis of mitochondrial membrane potential (MMP) in PC3 prostate cancer cells exposed to iridium complexes **Ir-TEMPO1** and **Ir-TEMPO2** at equipotent IC_{50} concentrations by flow cytometry using the JC-10 assay (Abcam).

References

1) O. V. Dolomanov, L. J. Bourhis, R. J. Gildea, J. A. K. Howard, H. Puschmann, *J. Appl. Cryst*., 2009, **42**, 339–341.
2) G. M. Sheldrick, *Acta Cryst. A*, 2008, **64**, 112–122.
3) G. M. Sheldrick, *Acta Cryst. C*, 2015, **71**, 3–8.
4) S. Stoll, A. Schweiger**,** *J. Magn. Reson.,* 2006, **178**, 42–55.
5) V. Venkatesh, C. J. Wedge, I. R. Canelón, A. Habtemariam and P. J. Sadler, *Dalton Trans.*, 2016, **45**, 13034–13037.
6) A. M. Brouwer, *Pure Appl. Chem*., 2011, **83**, 2213-2228.
7) J. Schindelin, I. Arganda-Carreras, E. Frise, V. Kaynig, M. Longair, T. Pietzsch, S. Preibisch, C. Rueden, S. Saalfeld, B. Schmid, J.-Y. Tinevez, D. J. White, V. Hartenstein, K. Eliceiri, P. Tomancak and A. Cardona, *Nat. Methods*, 2012, **9**, 676–682.
8) M. Mauro, G. D. Paoli, M. Otter, D. Donghi, G. D'Alfonso and L. D. Cola, *Dalton Trans*., 2011, **40**, 12106–12116.
9) R. J. Deeth, N.Fey, B. J. Williams-Hubbard, *J. Comput. Chem*., 2005, **26**, 123–130.
10) MOE; 2015.1001 ed.; Chemical Computing Group: Montreal, 2015.
11) T. A. Halgren, R. B. Nachbar, *J. Comput. Chem*., 1996, **17**, 587–615.
12) T. A. Halgren, *J. Comput. Chem*., 1996, **17**, 553–586.
13) T. A. Halgren, *J. Comput. Chem*., 1996, **17**, 520–552.
14) T. A. Halgren, *J. Comput. Chem*., 1996, **17**, 490–519.
15) R. Brodbeck, R. J. Deeth, *Dalton Trans*., 2011, **40**, 11147–11155.
